# The landscape of GWAS validation; systematic review identifying 309 validated non-coding variants across 130 human diseases

**DOI:** 10.1186/s12920-022-01216-w

**Published:** 2022-04-01

**Authors:** Ammar J. Alsheikh, Sabrina Wollenhaupt, Emily A. King, Jonas Reeb, Sujana Ghosh, Lindsay R. Stolzenburg, Saleh Tamim, Jozef Lazar, J. Wade Davis, Howard J. Jacob

**Affiliations:** 1 Genomics Research Center, AbbVie Inc, North Chicago, Illinois, 60064 USA; 2 Information Research, AbbVie Deutschland GmbH & Co. KG, 67061 Knollstrasse, Ludwigshafen, Germany

**Keywords:** GWAS, Experimental validation, Functional variant, Systematic review, Non-coding

## Abstract

**Background:**

The remarkable growth of genome-wide association studies (GWAS) has created a critical need to experimentally validate the disease-associated variants, 90% of which involve non-coding variants.

**Methods:**

To determine how the field is addressing this urgent need, we performed a comprehensive literature review identifying 36,676 articles. These were reduced to 1454 articles through a set of filters using natural language processing and ontology-based text-mining. This was followed by manual curation and cross-referencing against the GWAS catalog, yielding a final set of 286 articles.

**Results:**

We identified 309 experimentally validated non-coding GWAS variants, regulating 252 genes across 130 human disease traits. These variants covered a variety of regulatory mechanisms. Interestingly, 70% (215/309) acted through cis-regulatory elements, with the remaining through promoters (22%, 70/309) or non-coding RNAs (8%, 24/309). Several validation approaches were utilized in these studies, including gene expression (n = 272), transcription factor binding (n = 175), reporter assays (n = 171), in vivo models (n = 104), genome editing (n = 96) and chromatin interaction (n = 33).

**Conclusions:**

This review of the literature is the first to systematically evaluate the status and the landscape of experimentation being used to validate non-coding GWAS-identified variants. Our results clearly underscore the multifaceted approach needed for experimental validation, have practical implications on variant prioritization and considerations of target gene nomination. While the field has a long way to go to validate the thousands of GWAS associations, we show that progress is being made and provide exemplars of validation studies covering a wide variety of mechanisms, target genes, and disease areas.

**Supplementary Information:**

The online version contains supplementary material available at 10.1186/s12920-022-01216-w.

## Background

A central goal of genetics is to identify the genetic underpinnings of human diseases. Advancements in human genetics and its related fields and technologies over the past decades have had a remarkable impact on our understanding of human disease pathophysiology, diagnosis and management [1]. In Mendelian disorders and rare genetic diseases this often takes the form of a loss-of-function mutation or genomic abnormality driving the disease phenotype. There are more than 5,000 diseases that belong to this category accounted for in the Online Mendelian Inheritance in Man (OMIM) database [2]. For complex diseases, there are multiple genetic and environmental factors contributing to disease risk and the identification of genetic risk factors associated with complex diseases has been rapidly accelerating with the utilization of next generation sequencing and dense array genotyping technologies in genome-wide association studies (GWAS). In a GWAS, thousands of genetic variants are genotyped in individuals which are then used to identify statistical associations between variants at certain genomic loci and a particular phenotype [3]. Since the first reported GWAS association for age-related macular degeneration [4] the use of these studies have grown exponentially, with over 200,000 genetic variants associated with more than 3000 human traits reported [5]. The remarkable growth of GWAS has created a critical need to experimentally identify and validate the disease-associated variants [6, 7]. This barrier has hindered the translation of GWAS findings to disease biology mechanisms and hence therapies. There are seemingly very few examples of GWAS-identified genetic loci at which the causal variant and molecular mechanisms driving the association have been experimentally determined, especially considering the sheer number of genotype–phenotype associations that have been reported passing the genome-wide significance threshold.

Dissecting GWAS loci to uncover the underlying biology is a complicated multi-step process. High linkage disequilibrium (LD) between many variants often necessitates utilizing statistical fine-mapping approaches and overlapping with functional genomic annotations for prioritization of variants before experimental validation [3, 8]. For coding variants, the target gene is identified directly from the genomic location of the variant [9]. As protein-coding regions represent only a small percentage of the human genome, more than 90% of GWAS associated variants are annotated to be within non-coding parts of the genome [5]. Experimental identification and validation of non-coding variants involves additional level of complexity as compared to coding variants requiring the application of additional approaches [10, 11]. Moreover, the functionality of regulatory elements is often cell-type specific, which necessitates studying the mechanism in disease-relevant cell types [12].

Experimental identification and validation are critical elements in translating GWAS findings. To date there has been limited study of the number of GWAS-identified loci that have been experimentally validated. A systematic literature review of 36,676 published articles identified 309 experimentally validated non-coding GWAS variants, regulating 252 genes across 130 human disease traits. This review of the literature is the first to systematically evaluate the status and the landscape of experimentation being used to validate non-coding GWAS-identified variants. We additionally curated key information from all included studies such as validated variant class, distance-to-target gene, and experimental validation methods. Our findings have value for future experimental validation studies, target gene prioritization and functional variant prediction. The approaches utilized to validate coding variants as well as current methods used to nominate candidate functional variants for functional studies are outside the scope of this manuscript and have been reviewed previously [8, 9].

## Methods

We conducted a systematic literature search and report it in compliance with the standards set forth by the 2020 PRISMA statement on the reporting of systematic reviews [13]. As a traditional keyword-based search approach would not enable us to thoroughly search for all relevant concepts and combinations, we leveraged natural language processing (NLP) and ontology-based text mining to ensure a systematic identification of relevant validation articles [14, 15]. We defined the scope to include studies that perform validation of GWAS associated non-coding variants at least at a molecular level.

**Fig. 1:**
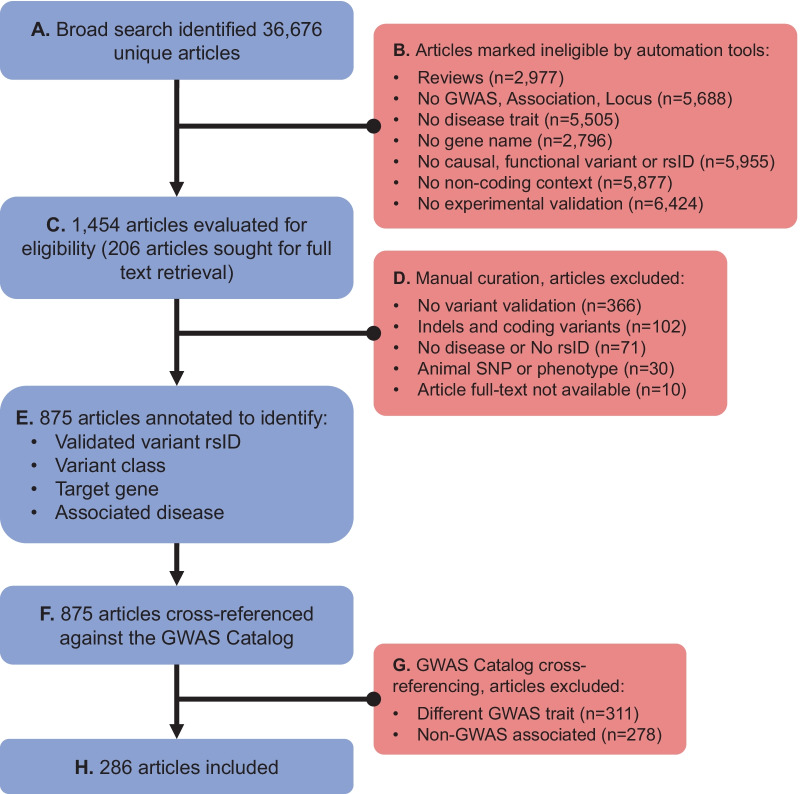
Systematic literature search and validation approach. Flow diagram demonstrating the systematic literature search strategy starting with **A** broad Medline search including all potentially related articles. The search included several concepts related to GWAS, non-coding contexts and other related terms detailed in Additional file 1. **B** Using text-mining of article titles, abstracts and metadata, we built seven filters to narrow down the search results which excluded 35,222 articles. Exact search terms and their combinations used in the filters are provided in Additional file 2. **C** 1454 articles of interest that passed all the filters were manually screened and evaluated for eligibility. **D** Through manual curation an additional set of 579 articles was excluded. **E** 875 eligible articles that passed manual curation were annotated to identify key information from each study. **F** These articles proceeded to cross-referencing against the GWAS Catalog to ensure that the validated variants and their reported associated disease trait match known GWAS associations. **G** Cross-referencing excluded 598 articles with poor GWAS trait matches or no variant match. **H** The final systematic review includes 286 articles. Reasons for exclusion at each stage are shown in red on the right side and described in more detail in the main text

In order to build a comprehensive literature search strategy, we first identified 28 validation studies from recent reviews and published resources [6, 7, 16]. These index studies were evaluated to identify the optimal keywords and concepts that would be used in the systematic literature search. Figure 1 shows a flow diagram summarizing the systematic literature search approach that was employed. The systematic literature search was conducted using search and filter concepts identified by thorough manual and text mining-supported concept analysis of index articles. The initial broad search was based on four different sub-queries aimed at identifying any articles that might include experimental validation of GWAS variants. We included explicit mention of GWAS, non-coding, functional or causal variant as well as contextual mentions of non-coding concepts such as enhancers and promoters (Additional file 1). Queries were run on MEDLINE Full Index [17] (all MEDLINE content until February 19, 2021) using IQVIA/Linguamatics I2E KNIME nodes [18]. Concepts and various combinations were searched in title, abstract and meta-data (author keywords, Medical Subject Headings (MeSH) terms and substances) leveraging public standard life science ontologies (such as MeSH [19], NCI Thesaurus [20] or Entrez Gene [21], custom vocabularies and syntactical rules, grammatical pattern and linguistic entity classes allowing to build more generalized (comprehensive) queries, but at the same time more precise queries than standard key word search engines. The PMIDs identified by each query were combined and filtered for publication year ≥ 2007 (using “PubMed Publication Data (entrez)”). After removing duplicates, we arrived at 36,676 unique articles (Fig. 1A). We built seven filters reflecting our key inclusion criteria to narrow down the search results: (1) filter for primary research articles and exclude other article types, (2) GWAS and/or association filter, (3) filter for any human disease, (4) filter for any human gene (RefSeq), (5) filter for explicit mention of “non-coding” or non-coding context (enhancers, intron, non-coding, microRNA, etc.), (6) filter for functional, causal, or regulatory variant or specific rsID, and (7) wet-lab experimental validation techniques (Fig. 1B, Additional file 2). Filters were built using an in-house entity extraction and literature classification pipeline combining SciBite’s TERMite (TERM identification, tagging & extraction) API coupled with SciBite’s VOCabs [22] and IQVIA/Linguamatics I2E Software.

In total 1454 articles passed all filter criteria and were then manually reviewed by three curators (Fig. 1C). All articles had to meet the following criteria to be considered for inclusion: (1) investigate variants associated with a human disease, (2) include experimental wet-lab molecular validation of one or more variants, (3) include putative validation of at least one non-coding variant, and (4) investigate single nucleotide polymorphisms (SNPs), excluding indels, purely coding, somatic, or rare variants. Abstracts and full texts were reviewed resulting in the exclusion of 579 articles (Fig. 1D). Overall, this manual review identified 875 potentially relevant articles. All these articles were manually curated to confirm the rsID of the reportedly validated variants, variant class, the reported regulated gene, and the associated disease (Fig. 1E).

We then used the information on the validated variant’s rsID and disease trait to cross validate our data with the GWAS Catalog [5] (accessed Mar 25, 2021) to confirm that each curated variant-disease association is reported in a GWAS (Fig. 1F). Corresponding associations were identified through LD between the curated SNP and the reported GWAS Catalog SNP, and similarity between the reported GWAS trait and the traits extracted from the PubMed abstract as detailed below. Because the GWAS Catalog only reports the lead variant for each locus, and this variant is not necessarily identical to the causal variant for the association, we performed an LD expansion from each top SNP to identify additional possible causal variants. Broad ancestry as reported in the GWAS Catalog was mapped to a 1000 Genomes superpopulation following methods we described recently [23]. For each associated SNP in the GWAS Catalog, an LD expansion was performed to identify SNPs within 1 Mb with LD r^2^ ≥ 0.5 in the corresponding 1000 Genomes super-population. A minor allele count threshold of 5 within the corresponding superpopulation was applied to reduce the impact of high variance LD estimates for rare variants. If it was not possible to map to a single superpopulation, LD expansion was performed using the full 1000 Genomes Phase 3 GRCh38 liftover to match the build used in the GWAS Catalog [24]. When the GWAS Catalog reported a specific risk allele, our LD expansion took this into account, such that for multiallelic SNPs we would only identify variants correlated with the reported allele. The choice of LD threshold is motivated by the goal to capture GWAS associations that could plausibly be explained by the cataloged variant and has been used elsewhere[25]. Using this methodology, it was possible to perform LD expansion for 91% of variants in the GWAS Catalog. GWAS Catalog variants for which an LD expansion was not possible were still included in the analysis but could only be matched to the reported variant rather than other possible causal variants.

GWAS Catalog Experimental Factor Ontology (EFO) terms and disease terms curated from the literature were mapped to the 2020 MeSH thesaurus vocabulary using the approach outlined previously [26]. To allow for inexact matches in MeSH terms (e.g., hypertension and systolic blood pressure), we use two similarity metrics: Lin-Resnik average similarity with a cutoff value of 0.75 [26, 27] and odds ratio of MeSH term co-occurrence in the same PubMed article with a cutoff of 20 [23]. We count a match between an article identified in our systematic review and a GWAS study if any GWAS Catalog association satisfies the following criteria: (1) The reported variant in the GWAS Catalog has LD R^2^ ≥ 0.5 to at least one curated variant, and (2) the reported trait in the GWAS Catalog has similarity to a main or manually curated disease from the PubMed abstract, meeting or exceeding the cutoff value. We excluded 347 SNPs in 311 articles from the analysis due to not being linked to a GWAS Catalog SNP. A further 292 SNPs contained within 278 articles were excluded due to a poor match between the reported GWAS trait and the trait reported in the abstract (Fig. 1G). The final curated catalog includes 286 articles (Fig. 1H) [28–313].

## Results

### Curated catalog of 309 validated GWAS non-coding variants

**Fig. 2:**
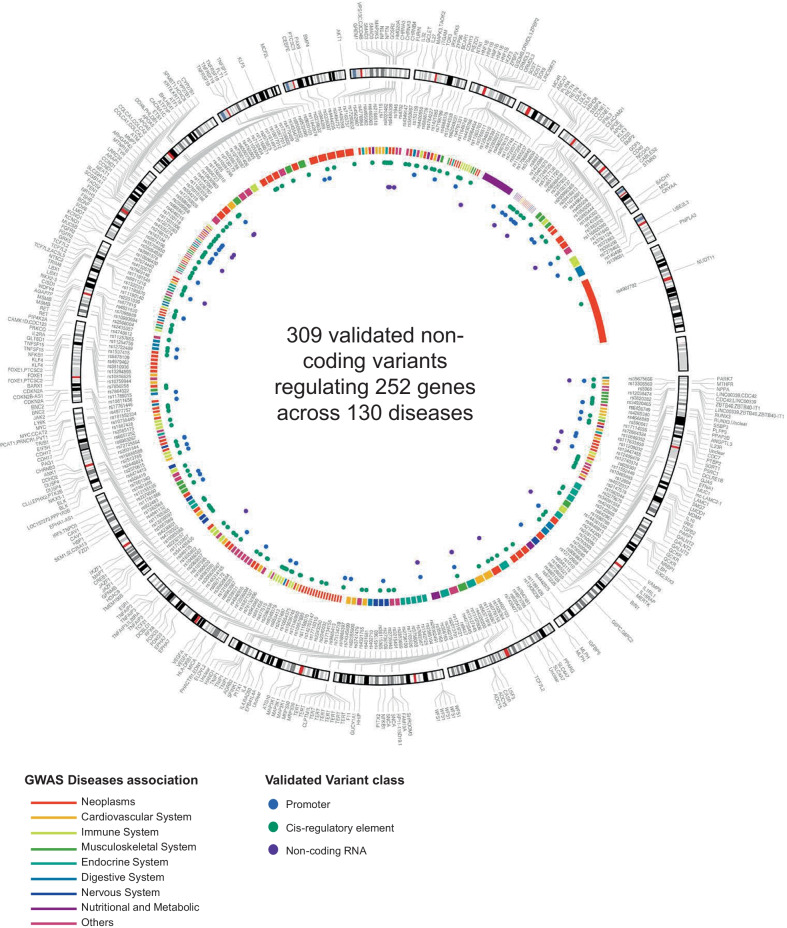
Map of 309 validated GWAS non-coding variants. The Circos plot displays the 309 experimentally validated variants studied within the 286 included articles. The outer most layer (i) shows the validated variants’ 252 target genes, (ii) the chromosomal map, (iii) the location of validated variants marked by their rsIDs, (iv) using higher order ontology mapping, we display inner links between variants associated with diseases in the same category. Disease systems that contain ten or more validated variants are displayed while those contain less than ten validated variants are grouped in “Others” category, and (v) the manually annotated validated variant class. Additional File 3 contains all variant details and annotations

**Fig. 3:**
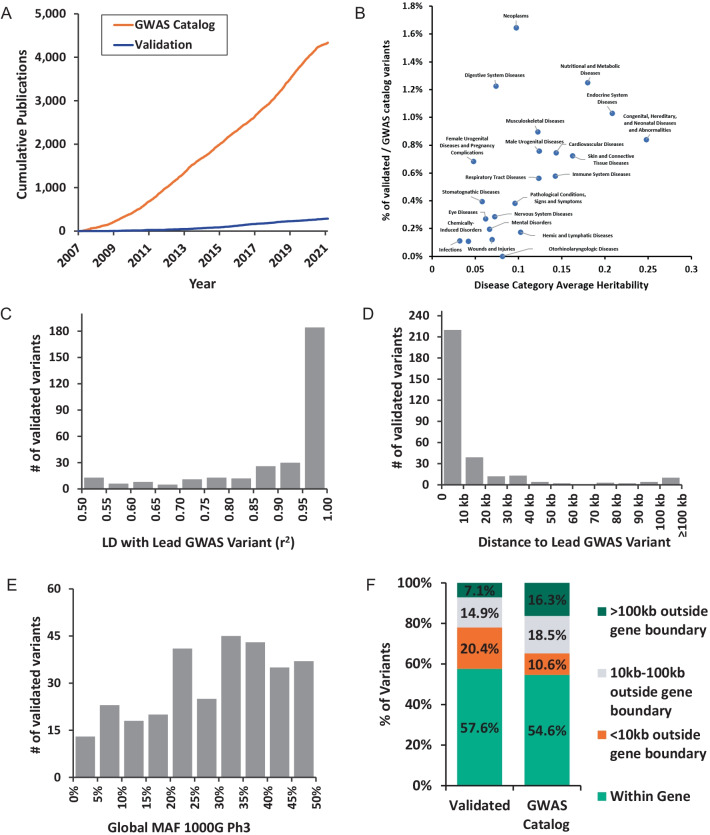
Functional validation remains the bottleneck of GWAS follow-up. **A** Comparison of the number of published studies in the GWAS catalog and non-coding variant validation studies over time. **B** Relationship between the ratio of validated non-coding variants to the total GWAS variants and disease category mean heritability. **C** Linkage disequilibrium between reported variant in GWAS Catalog and validated variants. **D** Distance between validated variant and GWAS Catalog-reported variant. **E** Global minor allele frequency (MAF) of validated variants in 1000 genomes phase 3. **F** Location of experimentally validated non-coding GWAS variants in relation to all protein-coding genes compared to GWAS lead variants

Several prior studies have emphasized the importance of experimental validations to uncover the biological processes underlying the statistical GWAS associations [3, 6, 7, 314, 315]. The final list of 286 articles reports 309 experimentally validated functional non-coding variants regulating 252 genes across 130 human-diseases (Additional file 3 and Fig. 2). Additional File 3 includes several important aspects about the included articles and variants including PubMed identifiers (PMID), variant rsID, location, class, target gene as well as disease associations and experimental validation approaches. We examined several characteristics of the validated non-coding variants in relation to GWAS catalog studies and variants. Between 2007 and 2020 there is a steady increase in the number of validation articles over time up to the 286 we report here. In contrast, the total number of published GWAS articles is 4342 versus 286 validation articles for non-coding variants (Fig. 3A). Next, we evaluated the relationship between disease heritability explained by common SNPs and the ratio of validated variants to the total number of lead-GWAS variants. We mapped disease associations for all variants to the higher order disease categories in the MeSH terms tree structure. For heritability estimates, we considered liability scale *h*^*2*^ for UK Biobank phenotypes estimated using LD Score Regression[316, 317] which (1) mapped to a MeSH disease (2) were considered high or medium confidence and averaged the heritability across higher level MeSH to get average heritability per disease category. Using this approach, we find a statistically significant (*p* = 0.01; correlation coefficient 0.51) positive relationship between mean heritability and the ratio of validated/lead GWAS variants per disease category (Fig. 3B). Examination of individual validated variants showed the majority of validated variants are in strong LD with and in close proximity to the GWAS variant (Fig. 3C, D). Allele frequencies of validated variants have slightly skewed distribution with fewer validated variants having lower allele frequencies (Fig. 3E). Comparing the location of experimentally validated non-coding GWAS variants to GWAS lead variants, we found that validated variants are about equally likely to be located within a protein-coding gene (58% for functional variants versus 55% for GWAS lead variants). However, they are much more likely to be within 10 kb of a gene boundary (20% versus 11%) and much less likely to be more than 100 kb from the nearest gene (7% versus 16%) (Fig. 3F). Overall, these findings quantify the persistent need for more experimental validation studies to bridge the gap between association and biology. These findings also suggest that focusing experimental validation efforts to variants in close proximity and strong LD to the lead GWAS variant would lead to the identification of a causal variant in the majority of genetic loci.

### Validated variants regulate 252 target genes through a variety of mechanisms

**Fig. 4:**
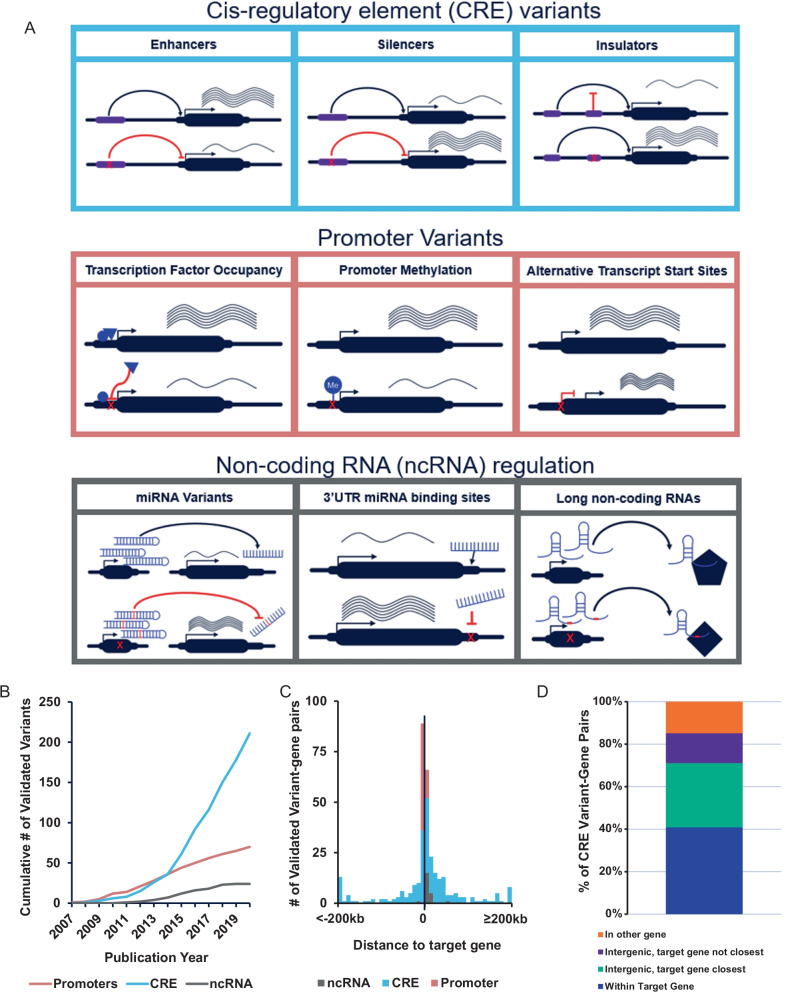
Non-coding variants regulate 252 target genes through diverse mechanisms. **A** Illustration of some of the diverse mechanisms of regulation within each variant category. Examples of each mechanism from included studies are discussed in the text. **B** Cumulative number of validated variants grouped by non-coding variant categories over time. **C** We used Encode’s Biomart and hg38 to calculate the distance (in kb) between validated variants and their target gene’s closest transcription start site (TSS). Graph plots the number of variant- gene pairs grouped by variant class. Variants more than 200 kb away are plotted at 200 kb. **D** Distribution of CRE variants relative to their target gene. CRE = Cis-Regulatory Element, ncRNA = non-coding RNA

Non-coding genetic variants can exert their effect on target genes through a variety of mechanisms [318–320]. We divided variants into three broad categories based on their mechanism of regulation: cis-regulatory element (CRE) variants, promoter variants and variants acting through non-coding RNAs (Fig. 4A). Promoter variants were grouped separately from other CREs because they are functionally distinct and in addition the methods utilized for their validation are different from other CREs. Below we highlight several exemplar studies validating variants across all these mechanisms and many diseases. Interestingly, the majority of non-coding variants identified in our catalog regulate genes through CREs (n = 215). These include variants in enhancers such as rs4420550-*MAPK3-TAOK2* in schizophrenia [168], rs11236797-*LRRC32* in inflammatory bowel disease [40], and rs9349379-*EDN1* in vascular diseases [49]. Some variants exerted their effect through silencers such as rs12038474-*CDC42* in endometriosis [130], rs2494737-*AKT1* in endometrial carcinoma [37] and rs9508032-*FLT1* in acute respiratory distress syndrome[267]. Additionally, rs12936231-*GSDMB-ORMDL3-ZPBP2* seems to function through an insulator in an asthma and autoimmune disease risk locus [71].

Variants in gene promoters can alter transcription factor binding and promoter activity. For example, rs1887428-*JAK2* in inflammatory bowel disease [256], rs11789015-*BARX1* in esophageal adenocarcinoma [88], rs4065275-*ORMDL3* and rs8076131-*ORMDL3* in asthma, [248] and rs11603334-*ARAP1* in type 2 diabetes mellitus [34]. DNA methylation is an important epigenetic mechanism of gene regulation and increased DNA methylation at gene promoters can repress gene transcription [321, 322]. We identified several validated variants that appear to alter promoter methylation including rs780093-*NRBP1* in gout [127], rs143383-*GDF5* in osteoarthritis [119], and rs35705950-*MUC5B* in idiopathic pulmonary fibrosis [258]. Alternatively, variants could alter promoter and transcription start site usage. Examples for these mechanisms in our catalog include rs922483*-BLK* in systemic lupus erythematosus [302] and rs10465885-*GJA5* in atrial fibrillation [32].

The third broad category by which variants from our catalog exert their regulatory effect is through non-coding RNAs [323]. microRNAs are a major and well-studied class of regulatory small non-coding RNAs. Variants in microRNAs are known to impact disease biology through post-transcriptional regulation of their target genes, primarily via 3’ untranslated region (UTR) binding [324–326]. GWAS variants located within microRNAs can alter their biogenesis, expression levels and/or target specificity, while variants located in target genes are capable of altering microRNA binding sites [326]. Examples of validated variants within microRNAs included in this catalog are miR-196a2 variant rs11614913 regulating *SFMBT1* and *HOXC8* in metabolic syndrome [277], and miR-4513 variant rs2168518 regulating GOSR2 in cardiometabolic diseases [51]. Given that microRNAs typically target hundreds to thousands of genes, it is very difficult to confidently assign target genes that are mediating the effect of a microRNA variant. On the other hand, studying variants located within mircoRNA-binding sites of target genes may yield more success in assigning underlying mechanisms [326, 327]. There are numerous examples of such variants reported in this catalog, such as rs5068 altering regulation of *NPPA* by miR-425 in hypertension [96], rs1058205 altering regulation of *KLK3* by miR-3162-5p and rs1010 altering regulation of *VAMP8* by miR-370 in prostate cancer [54], and rs372883 altering *BACH1* regulation by miR-1257 in pancreatic ductal adenocarcinoma [174]. Another important class of non-coding RNAs is long non-coding RNAs that are recognized to play an important role in biology and disease [328, 329]. Some examples of long non-coding RNA variants in this catalog include rs6983267 in *CCAT2* regulating cancer metabolism through allele-specific binding of *CPSF7* [76] and rs2147578 in *LAMC2-1* modulating microRNA binding to it in colorectal cancer [43]. We examined the distribution of these three broad categories of validated variants across publication dates. We observed a steady increase in the validation of promoter variants (n = 70) and variants acting through non-coding RNAs (n = 24) since 2007, but a sharp increase in the number of studies validating CRE variants around 2015. This trend persisted through 2020 to reach a total of 215 variants representing 70% of this catalog (Fig. 4B). We also characterized the distance between each validated variant and its target gene’s closest transcription start site according to variant category. As expected, promoter variants clustered immediately upstream or downstream of their target’s transcription start site. CRE variants were more widely distributed, but nevertheless, 157 (66%) of these fell within 50 kb from their target gene TSS. A notable example of a distally acting enhancer variant > 50 kb, is the obesity *FTO* locus variant rs1421085 regulating *IRX3* and *IRX5,* which are 500 kb and 1,163 kb away respectively [147]. Since the majority of variants acting through non-coding RNAs identified in our catalog were located within 3’ UTRs, this group of variants tended to cluster within 100 kb downstream of gene transcript start sites (Fig. 4C). The dataset gave us the opportunity to examine the relationship between CRE variants and their target genes (n = 235 CRE variant-target gene pairs). Plotting the distribution of CRE variants based on their location relative to the target gene indicated that 41% of CRE variants are located within their target gene, and an additional 30% are intergenic and their target gene is the closest gene to the variant. 14% of CRE variants were intergenic and their target gene is not the closest gene, and the remaining 15% are located within a different gene than their target gene. (Fig. 4D). These results are interesting and provide greater support for consideration of same gene and nearby genes as candidate targets for CREs. These findings are also in agreement with recent empirical data [330, 331].

**Fig. 5:**
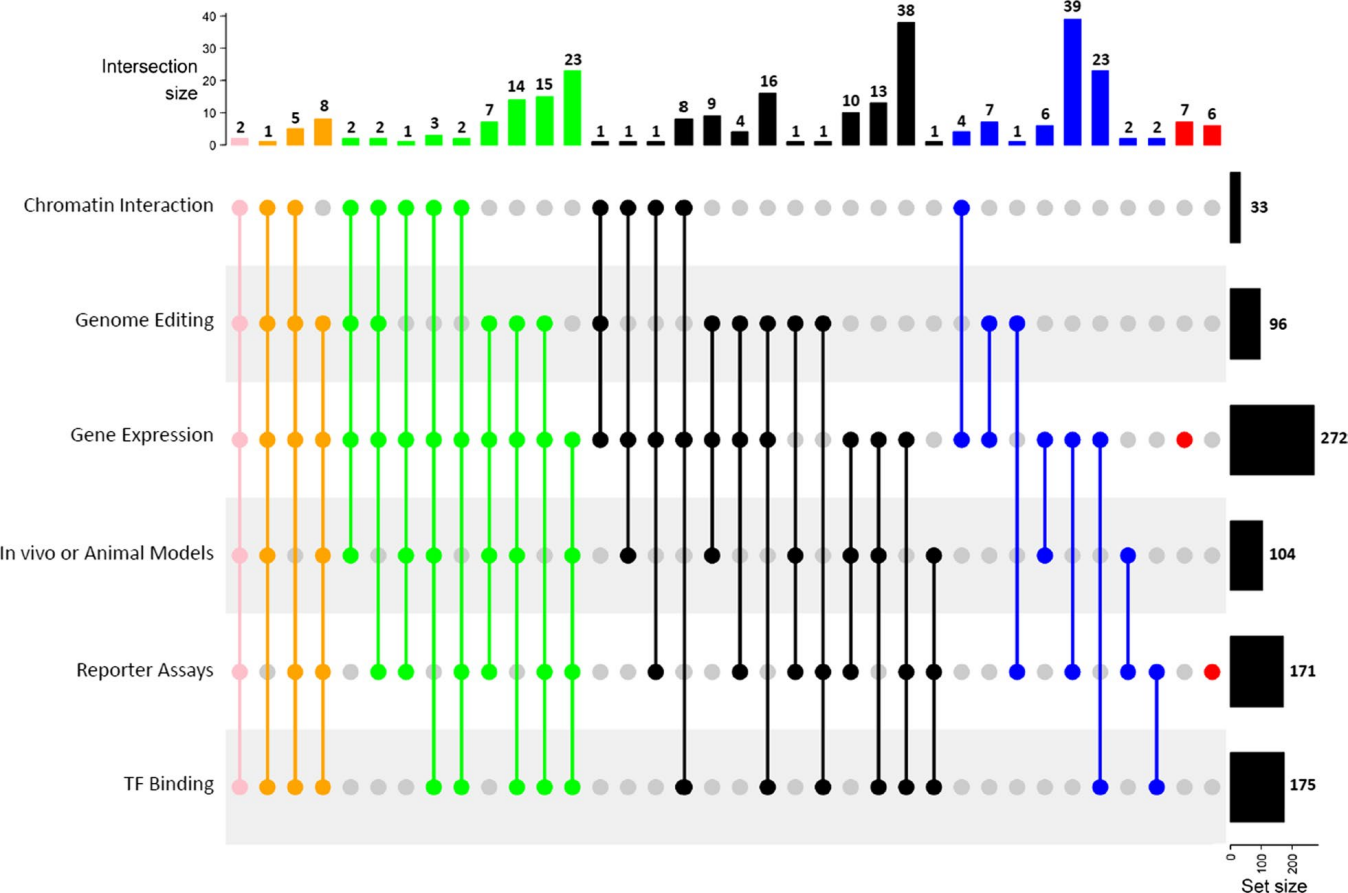
Studies utilize multiple avenues in validating non-coding variants. Using text-mining of abstracts and metadata, we examined the utilization of different avenues for non-coding variant validation across 286 included articles. The six broad categories were gene expression, reporter assays, transcription factor binding, in vivo or animal models, genome editing, and chromatin interaction. The intersection size denotes the number of articles that have the combination of validation categories below it. The color denotes the number of avenues used; pink – 6, orange—5, green—4, black—3, blue—2, red—1. The upset plot shows the overlap of the variant validation avenues and the number of articles. The Set size bars on the right reflect the total number of studies that used/employed each of the categories

Next, using text mining, we extracted and analyzed the experimental methods that were used in each study to validate variants. We broadly classified them under six broad categories covering different types of established validation techniques and related terms: (1) gene expression, including eQTL and molecular assessment of target gene expression and allele specific regulation (n = 272 articles), (2) reporter assays, including luciferase and massively parallel reporter assays (n = 171 articles), (3) transcription factor binding, including chromatin immunoprecipitation and electrophoretic mobility shift assays (n = 175 articles), (4) in vivo or animal models (n = 104 articles), (5) genome editing, including CRISPR and TALEN (n = 96 articles), and (6) chromatin interaction, including chromosome conformation capture (n = 33 articles) [11]. We examined the number of these approaches that were utilized by the included studies and found that 189 (66%) of all articles utilized three or more approaches (Fig. 5). These results demonstrate the multifaceted approach needed for validation of non-coding variants [11].

## Discussion

GWAS have seen a remarkable growth in the past decade. The impact of GWAS on human healthcare is severely limited by the bottle neck of experimental validation of disease-associated variants. Here, we report the first systematic approach to curate all experimental validation studies of non-coding GWAS variants. While there is general recognition that experimental validation of GWAS are seriously lacking [7], this systematic assessment of (1) the number of published experimentally validated non-coding variants is quantified, (2) cataloged, and (3) methods used in identified studies analyzed.

Using a comprehensive approach, we employed natural-language processing-based text mining, manual curation and GWAS catalog cross validation. We have curated 286 validation studies that include 309 putatively validated variants regulating 252 genes across 130 diseases. We then evaluated several important characteristics of the identified variants and their relation to GWAS lead variants. The ratio of validated non-coding variants to total GWAS lead variants showed a positive correlation to the mean heritability of disease groups. This relationship could indicate greater success in validating variants in diseases with higher heritability perhaps because of greater individual contribution of these variants to the overall disease susceptibility. This could also potentially represent a greater interest of scientists to pursue validation of variants in more heritable diseases and with larger effect sizes, thus leading to greater proportion of variants being validated. However, we do not have enough data to directly address this possibility. We also evaluated the relationship in LD and distance between validated variants and GWAS lead variants. We find that ~ 70% of validated variants fall within 10 kb and r^2^ ≥ 0.9 with the lead GWAS variant. On one hand, this could reflect underlying genetics that most validated variants are in strong LD with lead GWAS variants and suggests that more productive research should be limited to SNPs in high LD and closer distance to lead GWAS variants. On the other hand, the status quo might be reflective of prior limits in search space already considered by scientists who performed validation studies, however we do not have data to support this possibility[8].

Next, we annotated variants into broad classes based on the mechanisms by which these non-coding variants acted. This identified several interesting patterns, such as an increase in the number of variants functioning through cis-regulatory elements over time. One explanation for this increase could be the growing awareness of the importance of these regulatory elements in human biology and disease which has led to the initiation of large projects aimed at identification, annotation and prioritization of non-coding regulatory elements [10, 320, 332]. Additionally, several SNP-enrichment analyses have demonstrated that GWAS variants are significantly enriched in active regulatory regions [314]. We expect this trend to continue with publications by larger consortia and projects that investigate regulatory elements in different life stages, tissues and biological conditions [332]. Interestingly, the majority of cis-regulatory element variants that we found appeared to act through transcriptional enhancers. This dominance of enhancer variants over other regulatory elements might be a result of enhancer elements having more clearly defined functions and biochemical markers (i.e., histone modification signatures) [333, 334]. This highlights the potential for increased discovery of GWAS variants acting through silencers and insulators as our understanding of their distinct biochemical signatures is refined and assayed in disease relevant cell types [333, 335].

Our comprehensive search and filter strategy enabled us to identify validated variants across a large number of complex human diseases and those that act through a myriad of mechanisms. Nevertheless, the systematic search was limited to the MEDLINE database. Relevant articles published in journals not indexed in this standard database for biomedical literature will be missing in our data set [336, 337]. For quality control and to identify limitations of our search and filter approach, we analyzed the recall of our index studies throughout the entire process (Fig. 1A–H). It is important to highlight that broadening the initial search to include non-coding contexts and association/locus instead of limiting to explicit mentions of non-coding and GWAS terms ensured identification of relevant studies that we had otherwise missed. A significant number of index articles did not explicitly mention these terms [48, 78, 134, 143, 147, 171, 178, 210, 230, 256, 302]. Our final broad search covered 27 out of the 28 index studies which demonstrates good search coverage. Through an iterative process, we narrowed down these results, trying to maximize the recall of index studies while maintaining a manageable number of articles for manual review. We are aware that the implemented stringent criteria bias the search to exclude true validation articles that did not mention any disease, protein or specific experimental validation terms [338–345]. Additionally, the tagging of the articles and normalization of concepts for filtering relies on accurate named entity recognition (NER) and ontologies. Even when using highly curated, enriched vocabularies and state-of-the-art NER routines, recall rates of at maximum 80–95% are assumed (depending on entity type). Overall, a total of 19 index studies passed all filtering stages and were included in the final catalog. Finally, the data of our curated catalog is mainly based on the publications’ abstract information. Only in cases where information was missing or unclear in the abstract did we gather data from the full text. Therefore, it is possible that information gathered from the final set of articles may be incomplete. This would have affected the experimental validation techniques analysis in particular, which was based only on abstract mining.

Construction of the catalog using controlled vocabularies for diseases, variants, genes, variant classes, and functional follow up methods is aimed to facilitate use in bioinformatics follow up analyses. We expect this resource to be useful in evaluating the performance of computational fine mapping and target prioritization methods. Quantifying the performance of these methods on real datasets has previously been hindered by a lack of true positive examples. A large dataset of true positive examples would allow researchers to computationally identify features associated with functional variation. Recent efforts to compile such true positive datasets and use them to train target prioritization methods have come with concerns about bias towards coding variation [16] or are aimed at a specific trait subset such as molecular phenotypes [346] or immune disease [347]. We expect this catalog to contribute a large number of much needed examples of functional noncoding variants in human disease and the genes on which they act. Despite this important contribution, bias towards nearby genes and variants to the top GWAS SNP is still a concern for our catalog due to the limited number of variants and genes evaluated in the cataloged studies. To generate an unbiased training set for computational methods, an ideal functional study following up on a GWAS association would consider all credible causal SNPs and their nearby genes, but studies in our catalog typically consider a more limited set of genes and SNPs. For example, eQTL variants may be shared among multiple transcripts [348], and in this scenario functional studies considering only a single gene could be misleading about the causal gene.

## Conclusions

This review is the first to systematically evaluate the status and the landscape of experimentation being used to validate non-coding GWAS-identified variants. Our results clearly underscore the multifaceted approach needed for experimental validation. The findings of validated variants relationship to lead GWAS variants as well as to their target genes provide practical insights for future validation studies. Finally, we aim for the catalog to be a useful resource aiding in the development of prediction tools by providing a truth set of experimentally validated variants. Collectively this contributes to the overall effort to bridge the gap between genetic association and function in complex diseases.

## Supplementary Information

**Additional file 1** contains exact search terms and criteria used for creating the initial broad literature search.

**Additional file 2** contains exact terms and phrases used to setup the seven filters that were used to narrow down the broad search results.

**Additional file 3** contains all the validated variants and their details. The file is formatted to include separate rows for unique PMID-variant-gene triples, therefore variants that regulate multiple genes and variants that have been validated in more than one publication have more than one row in the file.

## Data Availability

The data supporting the conclusions of this article is included within the article (and its additional files).

## Abbreviations

CRE: Cis-regulatory element
GWAS: Genome-Wide Association Study
LD: Linkage disequilibrium
MeSH: Medical subject headings
PRISMA: Preferred reporting items for systematic reviews and meta-analyses
SNP: Single nucleotide polymorphism

## References

[1] Collins FS, Doudna JA, Lander ES, Rotimi CN (2021). Human molecular genetics and genomics—Important advances and exciting possibilities. N Engl J Med.

[2] OMIM - Online Mendelian Inheritance in Man. https://www.omim.org/. 2021 [cited 2021 Apr 11]; Available from: https://www.omim.org/

[3] Tam V, Patel N, Turcotte M, Bossé Y, Paré G, Meyre D (2019). Benefits and limitations of genome-wide association studies. Nat Rev Genet.

[4] Klein RJ, Zeiss C, Chew EY, Tsai J-Y, Sackler RS, Haynes C (2005). Complement factor H polymorphism in age-related macular degeneration. Science.

[5] Buniello A, MacArthur JAL, Cerezo M, Harris LW, Hayhurst J, Malangone C (2019). The NHGRI-EBI GWAS Catalog of published genome-wide association studies, targeted arrays and summary statistics 2019. Nucleic Acids Res.

[6] Visscher PM, Wray NR, Zhang Q, Sklar P, McCarthy MI, Brown MA (2017). 10 Years of GWAS discovery: biology, function, and translation. Am J Human Genet.

[7] Gallagher MD, Chen-Plotkin AS (2018). The Post-GWAS Era: From Association to Function. Am J Hum Genet.

[8] Schaid DJ, Chen W, Larson NB (2018). From genome-wide associations to candidate causal variants by statistical fine-mapping. Nat Rev Genet.

[9] Cai M, Ran D, Zhang X (2019). Advances in identifying coding variants of common complex diseases. J Bio-X Res.

[10] Tak YG, Farnham PJ (2015). Making sense of GWAS: using epigenomics and genome engineering to understand the functional relevance of SNPs in non-coding regions of the human genome. Epigenetics Chromatin.

[11] Rao S, Yao Y, Bauer DE (2021). Editing GWAS: experimental approaches to dissect and exploit disease-associated genetic variation. Genome Med.

[12] Liu B, Montgomery SB (2020). Identifying causal variants and genes using functional genomics in specialized cell types and contexts. Hum Genet.

[13] Page MJ, McKenzie JE, Bossuyt PM, Boutron I, Hoffmann TC, Mulrow CD (2021). The PRISMA 2020 statement: an updated guideline for reporting systematic reviews. BMJ: Br Med J Publ Group.

[14] Chang M, Chang M, Reed JZ, Milward D, Xu JJ, Cornell WD (2016). Developing timely insights into comparative effectiveness research with a text-mining pipeline. Drug Discov Today.

[15] McEntire R, Szalkowski D, Butler J, Kuo MS, Chang M, Chang M (2016). Application of an automated natural language processing (NLP) workflow to enable federated search of external biomedical content in drug discovery and development. Drug Discov Today.

[16] Ghoussaini M, Mountjoy E, Carmona M, Peat G, Schmidt EM, Hercules A (2021). Open Targets Genetics: systematic identification of trait-associated genes using large-scale genetics and functional genomics. Nucleic Acids Res.

[17] MEDLINE. http://wayback.archive-it.org/org-350/20180312141554/https://www.nlm.nih.gov/pubs/factsheets/medline.html. 2021 [cited 2021 Jun 15]; Available from: http://wayback.archive-it.org/org-350/20180312141554/https://www.nlm.nih.gov/pubs/factsheets/medline.html

[18] Linguamatics. https://www.linguamatics.com/. 2021 [cited 2021 Jun 15]; Available from: https://www.linguamatics.com/

[19] Medical Subject Headings - Home Page [Internet]. U.S. National Library of Medicine; [cited 2021 Jun 15]. Available from: https://www.nlm.nih.gov/mesh/meshhome.html

[20] NCI Thesaurus. https://ncit.nci.nih.gov/ncitbrowser/. 2021;

[21] NCBI Gene Database. https://www.ncbi.nlm.nih.gov/gene/. 2021 [cited 2021 Jun 15]; Available from: https://www.ncbi.nlm.nih.gov/gene/

[22] TERMite - SciBite. https://www.scibite.com/platform/termite/. SciBite [Internet]. 2021 [cited 2021 Jun 15]; Available from: https://www.scibite.com/platform/termite/

[23] King EA, Dunbar F, Davis JW, Degner JF (2021). Estimating colocalization probability from limited summary statistics. BMC Bioinform.

[24] Auton A, Abecasis GR, Altshuler DM, Durbin RM, Abecasis GR, Bentley DR (2015). A global reference for human genetic variation. Nature.

[25] Farh KK-H, Marson A, Zhu J, Kleinewietfeld M, Housley WJ, Beik S (2015). Genetic and epigenetic fine mapping of causal autoimmune disease variants. Nature.

[26] King EA, Davis JW, Degner JF (2019). Are drug targets with genetic support twice as likely to be approved? Revised estimates of the impact of genetic support for drug mechanisms on the probability of drug approval. PLoS Genet.

[27] Nelson MR, Tipney H, Painter JL, Shen J, Nicoletti P, Shen Y (2015). The support of human genetic evidence for approved drug indications. Nat Genet.

[28] Almontashiri NAM, Antoine D, Zhou X, Vilmundarson RO, Zhang SX, Hao KN (2015). 9p21.3 coronary artery disease risk variants disrupt TEAD transcription factor-dependent transforming growth factor β regulation of p16 expression in human aortic smooth muscle cells. Circulation.

[29] Yu C-Y, Han J-X, Zhang J, Jiang P, Shen C, Guo F (2020). A 16q22.1 variant confers susceptibility to colorectal cancer as a distal regulator of ZFP90. Oncogene.

[30] Piao X, Yahagi N, Takeuchi Y, Aita Y, Murayama Y, Sawada Y (2018). A candidate functional SNP rs7074440 in TCF7L2 alters gene expression through C-FOS in hepatocytes. FEBS Lett England.

[31] Kretschmer A, Möller G, Lee H, Laumen H, von Toerne C, Schramm K (2014). A common atopy-associated variant in the Th2 cytokine locus control region impacts transcriptional regulation and alters SMAD3 and SP1 binding. Allergy Denmark.

[32] Wirka RC, Gore S, Van Wagoner DR, Arking DE, Lubitz SA, Lunetta KL (2011). A common connexin-40 gene promoter variant affects connexin-40 expression in human atria and is associated with atrial fibrillation. Circ Arrhythm Electrophysiol.

[33] Lattka E, Eggers S, Moeller G, Heim K, Weber M, Mehta D (2010). A common FADS2 promoter polymorphism increases promoter activity and facilitates binding of transcription factor ELK1. J Lipid Res.

[34] Kulzer JR, Stitzel ML, Morken MA, Huyghe JR, Fuchsberger C, Kuusisto J (2014). A common functional regulatory variant at a type 2 diabetes locus upregulates ARAP1 expression in the pancreatic beta cell. Am J Hum Genet.

[35] Choi J, Xu M, Makowski MM, Zhang T, Law MH, Kovacs MA (2017). A common intronic variant of PARP1 confers melanoma risk and mediates melanocyte growth via regulation of MITF. Nat Genet United States.

[36] Kycia I, Wolford BN, Huyghe JR, Fuchsberger C, Vadlamudi S, Kursawe R (2018). A common Type 2 diabetes risk variant potentiates activity of an evolutionarily conserved islet stretch enhancer and increases C2CD4A and C2CD4B expression. Am J Hum Genet.

[37] Painter JN, Kaufmann S, O’Mara TA, Hillman KM, Sivakumaran H, Darabi H (2016). A common variant at the 14q32 endometrial cancer risk locus activates AKT1 through YY1 binding. Am J Hum Genet.

[38] Guo X, Lin W, Bao J, Cai Q, Pan X, Bai M (2018). A Comprehensive cis-eQTL analysis revealed target genes in breast cancer susceptibility loci identified in genome-wide association studies. Am J Hum Genet.

[39] Gallagher MD, Posavi M, Huang P, Unger TL, Berlyand Y, Gruenewald AL (2017). A dementia-associated risk variant near TMEM106B alters chromatin architecture and gene expression. Am J Hum Genet.

[40] Nasrallah R, Imianowski CJ, Bossini-Castillo L, Grant FM, Dogan M, Placek L (2020). A distal enhancer at risk locus 11q13.5 promotes suppression of colitis by T(reg) cells. Nature.

[41] Díaz-Jiménez D, Núñez L, De la Fuente M, Dubois-Camacho K, Sepúlveda H, Montecino M (2017). A functional IL1RL1 variant regulates corticosteroid-induced sST2 expression in ulcerative colitis. Sci Rep.

[42] Shou W, Wang Y, Xie F, Wang B, Yang L, Wu H (2014). A functional polymorphism affecting the APOA5 gene expression is causally associated with plasma triglyceride levels conferring coronary atherosclerosis risk in Han Chinese Population. Biochim Biophys Acta Netherlands.

[43] Gong J, Tian J, Lou J, Ke J, Li L, Li J (2016). A functional polymorphism in lnc-LAMC2-1:1 confers risk of colorectal cancer by affecting miRNA binding. Carcinogenesis England.

[44] Saeki N, Saito A, Choi IJ, Matsuo K, Ohnami S, Totsuka H (2011). A functional single nucleotide polymorphism in mucin 1, at chromosome 1q22, determines susceptibility to diffuse-type gastric cancer. Gastroenterol USA.

[45] Ogura Y, Kou I, Miura S, Takahashi A, Xu L, Takeda K (2015). A functional SNP in BNC2 is associated with adolescent idiopathic scoliosis. Am J Hum Genet.

[46] Ye J, Tucker NR, Weng L-C, Clauss S, Lubitz SA, Ellinor PT (2016). A functional variant associated with atrial fibrillation regulates PITX2c expression through TFAP2a. Am J Hum Genet.

[47] Akamatsu S, Takata R, Ashikawa K, Hosono N, Kamatani N, Fujioka T (2010). A functional variant in NKX3.1 associated with prostate cancer susceptibility down-regulates NKX3.1 expression. Hum Mol Genet.

[48] Ali MW, Patro CPK, Zhu JJ, Dampier CH, Plummer SJ, Kuscu C (2021). A functional variant on 20q13.33 related to glioma risk alters enhancer activity and modulates expression of multiple genes. Hum Mutat.

[49] Gupta RM, Hadaya J, Trehan A, Zekavat SM, Roselli C, Klarin D (2017). A genetic variant associated with five vascular diseases is a distal regulator of endothelin-1 gene expression. Cell.

[50] De Castro-Orós I, Pérez-López J, Mateo-Gallego R, Rebollar S, Ledesma M, León M (2014). A genetic variant in the LDLR promoter is responsible for part of the LDL-cholesterol variability in primary hypercholesterolemia. BMC Med Genomics.

[51] Ghanbari M, de Vries PS, de Looper H, Peters MJ, Schurmann C, Yaghootkar H (2014). A genetic variant in the seed region of miR-4513 shows pleiotropic effects on lipid and glucose homeostasis, blood pressure, and coronary artery disease. Hum Mutat USA.

[52] Stegeman S, Moya L, Selth LA, Spurdle AB, Clements JA, Batra J (2015). A genetic variant of MDM4 influences regulation by multiple microRNAs in prostate cancer. Endocr Relat Cancer England.

[53] Schaefer AS, Richter GM, Nothnagel M, Manke T, Dommisch H, Jacobs G (2010). A genome-wide association study identifies GLT6D1 as a susceptibility locus for periodontitis. Hum Mol Genet England.

[54] Stegeman S, Amankwah E, Klein K, O’Mara TA, Kim D, Lin H-Y (2015). A large-scale analysis of genetic variants within putative miRNA binding sites in prostate cancer. Cancer Discov.

[55] Kahali B, Chen Y, Feitosa MF, Bielak LF, O’Connell JR, Musani SK (2021). A noncoding variant near PPP1R3B promotes liver glycogen storage and MetS, but protects against myocardial infarction. J Clin Endocrinol Metab.

[56] Yan R, Lai S, Yang Y, Shi H, Cai Z, Sorrentino V (2016). A novel type 2 diabetes risk allele increases the promoter activity of the muscle-specific small ankyrin 1 gene. Sci Rep.

[57] Rodriguez BAT, Bhan A, Beswick A, Elwood PC, Niiranen TJ, Salomaa V (2020). A platelet function modulator of thrombin activation is causally linked to cardiovascular disease and affects PAR4 receptor signaling. Am J Hum Genet.

[58] Hing B, Davidson S, Lear M, Breen G, Quinn J, McGuffin P (2012). A polymorphism associated with depressive disorders differentially regulates brain derived neurotrophic factor promoter IV activity. Biol Psychiatry.

[59] Schieck M, Sharma V, Michel S, Toncheva AA, Worth L, Potaczek DP (2014). A polymorphism in the TH 2 locus control region is associated with changes in DNA methylation and gene expression. Allergy Denmark.

[60] Huang Q, Whitington T, Gao P, Lindberg JF, Yang Y, Sun J (2014). A prostate cancer susceptibility allele at 6q22 increases RFX6 expression by modulating HOXB13 chromatin binding. Nat Genet United States.

[61] Chang J, Tian J, Yang Y, Zhong R, Li J, Zhai K (2018). A Rare Missense variant in TCF7L2 associates with colorectal cancer risk by interacting with a GWAS-identified regulatory variant in the MYC enhancer. Cancer Res United States.

[62] Walavalkar K, Saravanan B, Singh AK, Jayani RS, Nair A, Farooq U (2020). A rare variant of African ancestry activates 8q24 lncRNA hub by modulating cancer associated enhancer. Nat Commun.

[63] Sinnott-Armstrong N, Sousa IS, Laber S, Rendina-Ruedy E, Nitter Dankel SE, Ferreira T (2021). A regulatory variant at 3q21.1 confers an increased pleiotropic risk for hyperglycemia and altered bone mineral density. Cell Metab.

[64] Chinnaswamy S, Chatterjee S, Boopathi R, Mukherjee S, Bhattacharjee S, Kundu TK (2013). A single nucleotide polymorphism associated with hepatitis C virus infections located in the distal region of the IL28B promoter influences NF-κB-mediated gene transcription. PLoS ONE.

[65] Lidral AC, Liu H, Bullard SA, Bonde G, Machida J, Visel A (2015). A single nucleotide polymorphism associated with isolated cleft lip and palate, thyroid cancer and hypothyroidism alters the activity of an oral epithelium and thyroid enhancer near FOXE1. Hum Mol Genet.

[66] Dos Santos C, Bougnères P, Fradin D (2009). A single-nucleotide polymorphism in a methylatable Foxa2 binding site of the G6PC2 promoter is associated with insulin secretion in vivo and increased promoter activity in vitro. Diabetes.

[67] Roman TS, Cannon ME, Vadlamudi S, Buchkovich ML, Wolford BN, Welch RP (2017). A Type 2 diabetes-associated functional regulatory variant in a pancreatic islet enhancer at the ADCY5 locus. Diabetes.

[68] Hiramoto M, Udagawa H, Ishibashi N, Takahashi E, Kaburagi Y, Miyazawa K (2018). A type 2 diabetes-associated SNP in KCNQ1 (rs163184) modulates the binding activity of the locus for Sp3 and Lsd1/Kdm1a, potentially affecting CDKN1C expression. Int J Mol Med.

[69] Justice CM, Kim J, Kim S-D, Kim K, Yagnik G, Cuellar A (2017). A variant associated with sagittal nonsyndromic craniosynostosis alters the regulatory function of a non-coding element. Am J Med Genet A.

[70] Jee SH, Sull JW, Lee J-E, Shin C, Park J, Kimm H (2010). Adiponectin concentrations: a genome-wide association study. Am J Hum Genet.

[71] Verlaan DJ, Berlivet S, Hunninghake GM, Madore A-M, Larivière M, Moussette S (2009). Allele-specific chromatin remodeling in the ZPBP2/GSDMB/ORMDL3 locus associated with the risk of asthma and autoimmune disease. Am J Hum Genet.

[72] Li X-X, Peng T, Gao J, Feng J-G, Wu D-D, Yang T (2019). Allele-specific expression identified rs2509956 as a novel long-distance cis-regulatory SNP for SCGB1A1, an important gene for multiple pulmonary diseases. Am J Physiol Lung Cell Mol Physiol.

[73] Palstra R-J, de Crignis E, Röling MD, van Staveren T, Kan TW, van Ijcken W (2018). Allele-specific long-distance regulation dictates IL-32 isoform switching and mediates susceptibility to HIV-1. Sci Adv.

[74] Benaglio P, D’Antonio-Chronowska A, Ma W, Yang F, Young Greenwald WW, Donovan MKR (2019). Allele-specific NKX2-5 binding underlies multiple genetic associations with human electrocardiographic traits. Nat Genet.

[75] Lee H, Qian K, von Toerne C, Hoerburger L, Claussnitzer M, Hoffmann C (2017). Allele-specific quantitative proteomics unravels molecular mechanisms modulated by cis-regulatory PPARG locus variation. Nucleic Acids Res.

[76] Redis RS, Vela LE, Lu W, Ferreira de Oliveira J, Ivan C, Rodriguez-Aguayo C, et al. Allele-specific reprogramming of cancer metabolism by the long non-coding RNA CCAT2. Mol Cell. 2016;61:520–34. 10.1016/j.molcel.2016.01.015 PMC4982398 26853146

[77] Richards TJ, Park C, Chen Y, Gibson KF, Di Peter Y, Pardo A (2012). Allele-specific transactivation of matrix metalloproteinase 7 by FOXA2 and correlation with plasma levels in idiopathic pulmonary fibrosis. Am J Physiol Lung Cell Mol Physiol.

[78] Fogarty MP, Panhuis TM, Vadlamudi S, Buchkovich ML, Mohlke KL (2013). Allele-specific transcriptional activity at type 2 diabetes-associated single nucleotide polymorphisms in regions of pancreatic islet open chromatin at the JAZF1 locus. Diabetes.

[79] Nakaoka H, Gurumurthy A, Hayano T, Ahmadloo S, Omer WH, Yoshihara K (2016). Allelic imbalance in regulation of ANRIL through chromatin interaction at 9p21 endometriosis risk locus. PLoS Genet.

[80] Pittman AM, Naranjo S, Jalava SE, Twiss P, Ma Y, Olver B (2010). Allelic variation at the 8q23.3 colorectal cancer risk locus functions as a cis-acting regulator of EIF3H. PLoS Genet.

[81] Barrie ES, Lee S-H, Frater JT, Kataki M, Scharre DW, Sadee W (2018). Alpha-synuclein mRNA isoform formation and translation affected by polymorphism in the human SNCA 3’UTR. Mol Genet Genomic Med.

[82] Gallego X, Cox RJ, Laughlin JR, Stitzel JA, Ehringer MA (2013). Alternative CHRNB4 3’-UTRs mediate the allelic effects of SNP rs1948 on gene expression. PLoS ONE.

[83] Wasserman NF, Aneas I, Nobrega MA (2010). An 8q24 gene desert variant associated with prostate cancer risk confers differential in vivo activity to a MYC enhancer. Genome Res.

[84] Thynn HN, Chen X-F, Hu W-X, Duan Y-Y, Zhu D-L, Chen H (2020). An allele-specific functional SNP associated with two systemic autoimmune diseases modulates IRF5 expression by long-range chromatin loop formation. J Invest Dermatol United States.

[85] Roberts AR, Vecellio M, Chen L, Ridley A, Cortes A, Knight JC (2016). An ankylosing spondylitis-associated genetic variant in the IL23R-IL12RB2 intergenic region modulates enhancer activity and is associated with increased Th1-cell differentiation. Ann Rheum Dis.

[86] Caussy C, Charrière S, Marçais C, Di Filippo M, Sassolas A, Delay M (2014). An APOA5 3’ UTR variant associated with plasma triglycerides triggers APOA5 downregulation by creating a functional miR-485-5p binding site. Am J Hum Genet.

[87] Wang S, Wen F, Wiley GB, Kinter MT, Gaffney PM (2013). An enhancer element harboring variants associated with systemic lupus erythematosus engages the TNFAIP3 promoter to influence A20 expression. PLoS Genet.

[88] Yan C, Ji Y, Huang T, Yu F, Gao Y, Gu Y (2018). An esophageal adenocarcinoma susceptibility locus at 9q22 also confers risk to esophageal squamous cell carcinoma by regulating the function of BARX1. Cancer Lett Ireland.

[89] Savic D, Bell GI, Nobrega MA (2012). An in vivo cis-regulatory screen at the type 2 diabetes associated TCF7L2 locus identifies multiple tissue-specific enhancers. PLoS ONE.

[90] Zhao H, Yang W, Qiu R, Li J, Xin Q, Wang X (2012). An intronic variant associated with systemic lupus erythematosus changes the binding affinity of Yinyang1 to downregulate WDFY4. Genes Immun England.

[91] Chen X-F, Zhu D-L, Yang M, Hu W-X, Duan Y-Y, Lu B-J (2018). An osteoporosis risk SNP at 1p36.12 acts as an allele-specific enhancer to modulate LINC00339 expression via long-range loop formation. Am J Hum Genet.

[92] Liu H, Duncan K, Helverson A, Kumari P, Mumm C, Xiao Y, et al. Analysis of zebrafish periderm enhancers facilitates identification of a regulatory variant near human KRT8/18. Elife. 2020;9. 10.7554/eLife.51325 PMC7039683 32031521

[93] Park JH, Chang HS, Park C-S, Jang A-S, Park BL, Rhim TY (2007). Association analysis of CD40 polymorphisms with asthma and the level of serum total IgE. Am J Respir Crit Care Med.

[94] Zhao Z, Fan Q, Zhou P, Ye H, Cai L, Lu Y (2017). Association of alpha A-crystallin polymorphisms with susceptibility to nuclear age-related cataract in a Han Chinese population. BMC Ophthalmol.

[95] De T, Alarcon C, Hernandez W, Liko I, Cavallari LH, Duarte JD (2018). Association of genetic variants with warfarin-associated bleeding among patients of African descent. JAMA.

[96] Arora P, Wu C, Khan AM, Bloch DB, Davis-Dusenbery BN, Ghorbani A (2013). Atrial natriuretic peptide is negatively regulated by microRNA-425. J Clin Invest.

[97] Gao P, Xia J-H, Sipeky C, Dong X-M, Zhang Q, Yang Y (2018). Biology and clinical implications of the 19q13 aggressive prostate cancer susceptibility locus. Cell.

[98] Bai X, Mangum KD, Dee RA, Stouffer GA, Lee CR, Oni-Orisan A (2017). Blood pressure-associated polymorphism controls ARHGAP42 expression via serum response factor DNA binding. J Clin Invest.

[99] de Smith AJ, Walsh KM, Francis SS, Zhang C, Hansen HM, Smirnov I (2018). BMI1 enhancer polymorphism underlies chromosome 10p12.31 association with childhood acute lymphoblastic leukemia. Int J Cancer.

[100] Cowper-Sal lari R, Zhang X, Wright JB, Bailey SD, Cole MD, Eeckhoute J, et al. Breast cancer risk-associated SNPs modulate the affinity of chromatin for FOXA1 and alter gene expression. Nat Genet. 2012;44:1191–8. 10.1038/ng.2416 PMC3483423 23001124

[101] Shah MY, Ferracin M, Pileczki V, Chen B, Redis R, Fabris L (2018). Cancer-associated rs6983267 SNP and its accompanying long noncoding RNA CCAT2 induce myeloid malignancies via unique SNP-specific RNA mutations. Genome Res.

[102] Glubb DM, Shi W, Beesley J, Fachal L, Pritchard J-L, McCue K, et al. Candidate Causal Variants at the 8p12 Breast Cancer Risk Locus Regulate DUSP4. Cancers (Basel). 2020;12. 10.3390/cancers12010170 PMC7016765 31936698

[103] McGovern A, Schoenfelder S, Martin P, Massey J, Duffus K, Plant D (2016). Capture Hi-C identifies a novel causal gene, IL20RA, in the pan-autoimmune genetic susceptibility region 6q23. Genome Biol.

[104] Ahluwalia TS, Troelsen JT, Balslev-Harder M, Bork-Jensen J, Thuesen BH, Cerqueira C (2017). Carriers of a VEGFA enhancer polymorphism selectively binding CHOP/DDIT3 are predisposed to increased circulating levels of thyroid-stimulating hormone. J Med Genet England.

[105] Spisák S, Lawrenson K, Fu Y, Csabai I, Cottman RT, Seo J-H (2015). CAUSEL: an epigenome- and genome-editing pipeline for establishing function of noncoding GWAS variants. Nat Med.

[106] Mehta ZB, Fine N, Pullen TJ, Cane MC, Hu M, Chabosseau P (2016). Changes in the expression of the type 2 diabetes-associated gene VPS13C in the β-cell are associated with glucose intolerance in humans and mice. Am J Physiol Endocrinol Metab.

[107] Prokop JW, Yeo NC, Ottmann C, Chhetri SB, Florus KL, Ross EJ (2018). Characterization of coding/noncoding variants for SHROOM3 in patients with CKD. J Am Soc Nephrol.

[108] Xia Q, Deliard S, Yuan C-X, Johnson ME, Grant SFA (2015). Characterization of the transcriptional machinery bound across the widely presumed type 2 diabetes causal variant, rs7903146, within TCF7L2. Eur J Hum Genet.

[109] Comiskey DFJ, He H, Liyanarachchi S, Sheikh MS, Hendrickson IV, Yu L (2020). Characterizing the function of EPB41L4A in the predisposition to papillary thyroid carcinoma. Sci Rep.

[110] Du M, Tillmans L, Gao J, Gao P, Yuan T, Dittmar RL (2016). Chromatin interactions and candidate genes at ten prostate cancer risk loci. Sci Rep.

[111] Matoba N, Liang D, Sun H, Aygün N, McAfee JC, Davis JE (2020). Common genetic risk variants identified in the SPARK cohort support DDHD2 as a candidate risk gene for autism. Transl Psychiatry.

[112] Hiramoto M, Udagawa H, Watanabe A, Miyazawa K, Ishibashi N, Kawaguchi M (2015). Comparative analysis of type 2 diabetes-associated SNP alleles identifies allele-specific DNA-binding proteins for the KCNQ1 locus. Int J Mol Med Greece.

[113] Hazelett DJ, Rhie SK, Gaddis M, Yan C, Lakeland DL, Coetzee SG (2014). Comprehensive functional annotation of 77 prostate cancer risk loci. PLoS Genet.

[114] Cheng M, Huang X, Zhang M, Huang Q (2020). Computational and functional analyses of T2D GWAS SNPs for transcription factor binding. Biochem Biophys Res Commun United States.

[115] Ye W, Wang Y, Mei B, Hou S, Liu X, Wu G (2018). Computational and functional characterization of four SNPs in the SOST locus associated with osteoporosis. Bone United States.

[116] Clifton-Bligh RJ, Nguyen TV, Au A, Bullock M, Cameron I, Cumming R (2011). Contribution of a common variant in the promoter of the 1-α-hydroxylase gene (CYP27B1) to fracture risk in the elderly. Calcif Tissue Int.

[117] Miller CL, Haas U, Diaz R, Leeper NJ, Kundu RK, Patlolla B (2014). Coronary heart disease-associated variation in TCF21 disrupts a miR-224 binding site and miRNA-mediated regulation. PLoS Genet.

[118] Gee F, Rushton MD, Loughlin J, Reynard LN (2015). Correlation of the osteoarthritis susceptibility variants that map to chromosome 20q13 with an expression quantitative trait locus operating on NCOA3 and with functional variation at the polymorphism rs116855380. Arthritis Rheumatol.

[119] Reynard LN, Bui C, Syddall CM, Loughlin J (2014). CpG methylation regulates allelic expression of GDF5 by modulating binding of SP1 and SP3 repressor proteins to the osteoarthritis susceptibility SNP rs143383. Hum Genet.

[120] Wu J, Yang S, Yu D, Gao W, Liu X, Zhang K (2019). CRISPR/cas9 mediated knockout of an intergenic variant rs6927172 identified IL-20RA as a new risk gene for multiple autoimmune diseases. Genes Immun England.

[121] Deng Y, Zhao J, Sakurai D, Sestak AL, Osadchiy V, Langefeld CD (2016). Decreased SMG7 expression associates with lupus-risk variants and elevated antinuclear antibody production. Ann Rheum Dis.

[122] Vezzoli G, Terranegra A, Aloia A, Arcidiacono T, Milanesi L, Mosca E (2013). Decreased transcriptional activity of calcium-sensing receptor gene promoter 1 is associated with calcium nephrolithiasis. J Clin Endocrinol Metab.

[123] Ryu J, Lee C (2016). Differential promoter activity by nucleotide substitution at a type 2 diabetes genome-wide association study signal upstream of the wolframin gene. J Diabetes Australia.

[124] Smith JG, Felix JF, Morrison AC, Kalogeropoulos A, Trompet S, Wilk JB (2016). Discovery of genetic variation on chromosome 5q22 associated with mortality in heart failure. PLoS Genet.

[125] Miller CL, Anderson DR, Kundu RK, Raiesdana A, Nürnberg ST, Diaz R (2013). Disease-related growth factor and embryonic signaling pathways modulate an enhancer of TCF21 expression at the 6q23.2 coronary heart disease locus. PLoS Genet.

[126] Rahimov F, Marazita ML, Visel A, Cooper ME, Hitchler MJ, Rubini M (2008). Disruption of an AP-2alpha binding site in an IRF6 enhancer is associated with cleft lip. Nat Genet.

[127] Zhu Z, Meng W, Liu P, Zhu X, Liu Y, Zou H (2017). DNA hypomethylation of a transcription factor binding site within the promoter of a gout risk gene NRBP1 upregulates its expression by inhibition of TFAP2A binding. Clin Epigenetics.

[128] Wang X, Srivastava Y, Jankowski A, Malik V, Wei Y, Del Rosario RC (2018). DNA-mediated dimerization on a compact sequence signature controls enhancer engagement and regulation by FOXA1. Nucleic Acids Res.

[129] Kim BS, Park S-M, Uhm TG, Kang JH, Park J-S, Jang A-S (2010). Effect of single nucleotide polymorphisms within the interleukin-4 promoter on aspirin intolerance in asthmatics and interleukin-4 promoter activity. Pharmacogenet Genomics United States.

[130] Powell JE, Fung JN, Shakhbazov K, Sapkota Y, Cloonan N, Hemani G (2016). Endometriosis risk alleles at 1p36.12 act through inverse regulation of CDC42 and LINC00339. Hum Mol Genet.

[131] Gant VU, Junco JJ, Terrell M, Rashid R, Rabin KR (2021). Enhancer polymorphisms at the IKZF1 susceptibility locus for acute lymphoblastic leukemia impact B-cell proliferation and differentiation in both Down syndrome and non-Down syndrome genetic backgrounds. PLoS ONE.

[132] Sio YY, Matta SA, Ng YT, Chew FT (2020). Epistasis between phenylethanolamine N-methyltransferase and β2-adrenergic receptor influences extracellular epinephrine level and associates with the susceptibility to allergic asthma. Clin Exp Allergy England.

[133] Vecellio M, Cortes A, Roberts AR, Ellis J, Cohen CJ, Knight JC (2018). Evidence for a second ankylosing spondylitis-associated RUNX3 regulatory polymorphism. RMD Open.

[134] Ghoussaini M, Edwards SL, Michailidou K, Nord S, Cowper-Sal Lari R, Desai K (2014). Evidence that breast cancer risk at the 2q35 locus is mediated through IGFBP5 regulation. Nat Commun.

[135] Shepherd C, Skelton AJ, Rushton MD, Reynard LN, Loughlin J (2015). Expression analysis of the osteoarthritis genetic susceptibility locus mapping to an intron of the MCF2L gene and marked by the polymorphism rs11842874. BMC Med Genet.

[136] Surgucheva I, Surguchov A (2011). Expression of caveolin in trabecular meshwork cells and its possible implication in pathogenesis of primary open angle glaucoma. Mol Vis.

[137] Lou H, Yeager M, Li H, Bosquet JG, Hayes RB, Orr N (2009). Fine mapping and functional analysis of a common variant in MSMB on chromosome 10q11.2 associated with prostate cancer susceptibility. Proc Natl Acad Sci USA.

[138] Chang B-L, Cramer SD, Wiklund F, Isaacs SD, Stevens VL, Sun J (2009). Fine mapping association study and functional analysis implicate a SNP in MSMB at 10q11 as a causal variant for prostate cancer risk. Hum Mol Genet.

[139] Westra H-J, Martínez-Bonet M, Onengut-Gumuscu S, Lee A, Luo Y, Teslovich N (2018). Fine-mapping and functional studies highlight potential causal variants for rheumatoid arthritis and type 1 diabetes. Nat Genet.

[140] Orr N, Dudbridge F, Dryden N, Maguire S, Novo D, Perrakis E (2015). Fine-mapping identifies two additional breast cancer susceptibility loci at 9q31.2. Hum Mol Genet.

[141] Painter JN, O’Mara TA, Batra J, Cheng T, Lose FA, Dennis J (2015). Fine-mapping of the HNF1B multicancer locus identifies candidate variants that mediate endometrial cancer risk. Hum Mol Genet.

[142] Pan Y, Tian R, Lee C, Bao G, Gibson G. Fine-mapping within eQTL credible intervals by expression CROP-seq. Biol Methods Protoc. 2020;5:bpaa008. 10.1093/biomethods/bpaa008 PMC7334875 32665975

[143] Glubb DM, Maranian MJ, Michailidou K, Pooley KA, Meyer KB, Kar S (2015). Fine-scale mapping of the 5q11.2 breast cancer locus reveals at least three independent risk variants regulating MAP3K1. Am J Hum Genet.

[144] Meyer KB, O’Reilly M, Michailidou K, Carlebur S, Edwards SL, French JD (2013). Fine-scale mapping of the FGFR2 breast cancer risk locus: putative functional variants differentially bind FOXA1 and E2F1. Am J Hum Genet.

[145] Cheng TH, Thompson DJ, O’Mara TA, Painter JN, Glubb DM, Flach S (2016). Five endometrial cancer risk loci identified through genome-wide association analysis. Nat Genet.

[146] Bohaczuk SC, Thackray VG, Shen J, Skowronska-Krawczyk D, Mellon PL. FSHB Transcription is Regulated by a Novel 5’ Distal Enhancer With a Fertility-Associated Single Nucleotide Polymorphism. Endocrinology. 2021;162. 10.1210/endocr/bqaa181 PMC7846141 33009549

[147] Claussnitzer M, Dankel SN, Kim K-H, Quon G, Meuleman W, Haugen C (2015). FTO obesity variant circuitry and adipocyte browning in humans. N Engl J Med.

[148] Buckley MA, Woods NT, Tyrer JP, Mendoza-Fandiño G, Lawrenson K, Hazelett DJ (2019). Functional analysis and fine mapping of the 9p22.2 ovarian cancer susceptibility locus. Cancer Res.

[149] Boardman-Pretty F, Smith AJP, Cooper J, Palmen J, Folkersen L, Hamsten A (2015). Functional analysis of a carotid intima-media thickness locus implicates BCAR1 and suggests a causal variant. Circ Cardiovasc Genet United States.

[150] Turner AW, Martinuk A, Silva A, Lau P, Nikpay M, Eriksson P (2016). Functional analysis of a novel genome-wide association study signal in SMAD3 that confers protection from coronary artery disease. Arterioscler Thromb Vasc Biol United States.

[151] Hamdi Y, Leclerc M, Dumont M, Dubois S, Tranchant M, Reimnitz G, et al. Functional analysis of promoter variants in genes involved in sex steroid action, DNA repair and cell cycle control. Genes (Basel). 2019;10. 10.3390/genes10030186 PMC6470759 30823486

[152] Pang DX, Smith AJP, Humphries SE (2013). Functional analysis of TCF7L2 genetic variants associated with type 2 diabetes. Nutr Metab Cardiovasc Dis.

[153] Baskin R, Woods NT, Mendoza-Fandiño G, Forsyth P, Egan KM, Monteiro ANA (2015). Functional analysis of the 11q23.3 glioma susceptibility locus implicates PHLDB1 and DDX6 in glioma susceptibility. Sci Rep.

[154] Egli RJ, Southam L, Wilkins JM, Lorenzen I, Pombo-Suarez M, Gonzalez A (2009). Functional analysis of the osteoarthritis susceptibility-associated GDF5 regulatory polymorphism. Arthritis Rheum.

[155] Douvris A, Soubeyrand S, Naing T, Martinuk A, Nikpay M, Williams A (2014). Functional analysis of the TRIB1 associated locus linked to plasma triglycerides and coronary artery disease. J Am Heart Assoc.

[156] Zhang Y, Kuipers AL, Yerges-Armstrong LM, Nestlerode CS, Jin Z, Wheeler VW (2010). Functional and association analysis of frizzled 1 (FZD1) promoter haplotypes with femoral neck geometry. Bone.

[157] Fang J, Jia J, Makowski M, Xu M, Wang Z, Zhang T (2017). Functional characterization of a multi-cancer risk locus on chr5p15.33 reveals regulation of TERT by ZNF148. Nat Commun.

[158] Eckart N, Song Q, Yang R, Wang R, Zhu H, McCallion AS (2016). Functional characterization of schizophrenia-associated variation in CACNA1C. PLoS ONE.

[159] Flora AV, Zambrano CA, Gallego X, Miyamoto JH, Johnson KA, Cowan KA (2013). Functional characterization of SNPs in CHRNA3/B4 intergenic region associated with drug behaviors. Brain Res.

[160] Bigot P, Colli LM, Machiela MJ, Jessop L, Myers TA, Carrouget J (2016). Functional characterization of the 12p12.1 renal cancer-susceptibility locus implicates BHLHE41. Nat Commun.

[161] Roca-Ayats N, Martínez-Gil N, Cozar M, Gerousi M, Garcia-Giralt N, Ovejero D (2019). Functional characterization of the C7ORF76 genomic region, a prominent GWAS signal for osteoporosis in 7q21.3. Bone.

[162] Kessler T, Wobst J, Wolf B, Eckhold J, Vilne B, Hollstein R (2017). Functional characterization of the GUCY1A3 coronary artery disease risk locus. Circulation.

[163] Maloney B, Ge Y-W, Petersen RC, Hardy J, Rogers JT, Pérez-Tur J (2010). Functional characterization of three single-nucleotide polymorphisms present in the human APOE promoter sequence: Differential effects in neuronal cells and on DNA-protein interactions. Am J Med Genet B Neuropsychiatr Genet.

[164] Helbig S, Wockner L, Bouendeu A, Hille-Betz U, McCue K, French JD (2017). Functional dissection of breast cancer risk-associated TERT promoter variants. Oncotarget.

[165] Ge M, Shi M, An C, Yang W, Nie X, Zhang J (2016). Functional evaluation of TERT-CLPTM1L genetic variants associated with susceptibility of papillary thyroid carcinoma. Sci Rep.

[166] Elsby LM, Orozco G, Denton J, Worthington J, Ray DW, Donn RP (2010). Functional evaluation of TNFAIP3 (A20) in rheumatoid arthritis. Clin Exp Rheumatol.

[167] Vecellio M, Chen L, Cohen CJ, Cortes A, Li Y, Bonham S (2021). Functional genomic analysis of a RUNX3 polymorphism associated with ankylosing spondylitis. Arthritis Rheumatol United States.

[168] Chang H, Cai X, Li H-J, Liu W-P, Zhao L-J, Zhang C-Y (2021). Functional genomics identify a regulatory risk variation rs4420550 in the 16p11.2 Schizophrenia-Associated Locus. Biol Psychiatry. United States.

[169] Guo L, Yamashita H, Kou I, Takimoto A, Meguro-Horike M, Horike S (2016). Functional Investigation of a Non-coding Variant Associated with Adolescent Idiopathic Scoliosis in Zebrafish: Elevated Expression of the Ladybird Homeobox Gene Causes Body Axis Deformation. PLoS Genet.

[170] Kong M, Kim Y, Lee C (2014). Functional investigation of a venous thromboembolism GWAS signal in a promoter region of coagulation factor XI gene. Mol Biol Rep Netherlands.

[171] Lawrenson K, Kar S, McCue K, Kuchenbaeker K, Michailidou K, Tyrer J (2016). Functional mechanisms underlying pleiotropic risk alleles at the 19p13.1 breast-ovarian cancer susceptibility locus. Nat Commun.

[172] Pérez-Razo JC, Cano-Martínez LJ, Vargas Alarcón G, Canizales-Quinteros S, Martínez-Rodríguez N, Canto P (2015). Functional polymorphism rs13306560 of the MTHFR gene is associated with essential hypertension in a Mexican-Mestizo Population. Circ Cardiovasc Genet United States.

[173] Nanda V, Wang T, Pjanic M, Liu B, Nguyen T, Matic LP (2018). Functional regulatory mechanism of smooth muscle cell-restricted LMOD1 coronary artery disease locus. PLoS Genet.

[174] Huang X, Zheng J, Li J, Che X, Tan W, Tan W (2018). Functional role of BTB and CNC Homology 1 gene in pancreatic cancer and its association with survival in patients treated with gemcitabine. Theranostics.

[175] Ustiugova AS, Korneev KV, Kuprash DV, Afanasyeva AMA. Functional SNPs in the Human Autoimmunity-Associated Locus 17q12–21. Genes (Basel). 2019;10. 10.3390/genes10020077 PMC6409600 30678091

[176] Klein JC, Keith A, Rice SJ, Shepherd C, Agarwal V, Loughlin J (2019). Functional testing of thousands of osteoarthritis-associated variants for regulatory activity. Nat Commun.

[177] Yu W, Zhang K, Wang Z, Zhang J, Chen T, Jin L (2017). Functional variant in the promoter region of IL-27 alters gene transcription and confers a risk for ulcerative colitis in northern Chinese Han. Hum Immunol United States.

[178] French JD, Ghoussaini M, Edwards SL, Meyer KB, Michailidou K, Ahmed S (2013). Functional variants at the 11q13 risk locus for breast cancer regulate cyclin D1 expression through long-range enhancers. Am J Hum Genet.

[179] Andiappan AK, Sio YY, Lee B, Suri BK, Matta SA, Lum J (2016). Functional variants of 17q12-21 are associated with allergic asthma but not allergic rhinitis. J Allergy Clin Immunol United States.

[180] Li Y, Nie Y, Cao J, Tu S, Lin Y, Du Y (2014). G-A variant in miR-200c binding site of EFNA1 alters susceptibility to gastric cancer. Mol Carcinog United States.

[181] Gaulton KJ, Ferreira T, Lee Y, Raimondo A, Mägi R, Reschen ME (2015). Genetic fine mapping and genomic annotation defines causal mechanisms at type 2 diabetes susceptibility loci. Nat Genet.

[182] Liu S, Wu N, Zuo Y, Zhou Y, Liu J, Liu Z, et al. Genetic Polymorphism of LBX1 Is Associated With Adolescent Idiopathic Scoliosis in Northern Chinese Han Population. Spine (Phila Pa 1976). United States; 2017;42:1125–9. 10.1097/BRS.0000000000002111 28187071

[183] Oldridge DA, Wood AC, Weichert-Leahey N, Crimmins I, Sussman R, Winter C (2015). Genetic predisposition to neuroblastoma mediated by a LMO1 super-enhancer polymorphism. Nature.

[184] Cavalli M, Pan G, Nord H, Wallén Arzt E, Wallerman O, Wadelius C (2017). Genetic prevention of hepatitis C virus-induced liver fibrosis by allele-specific downregulation of MERTK. Hepatol Res Netherlands.

[185] Krause MD, Huang R-T, Wu D, Shentu T-P, Harrison DL, Whalen MB (2018). Genetic variant at coronary artery disease and ischemic stroke locus 1p32.2 regulates endothelial responses to hemodynamics. Proc Natl Acad Sci USA.

[186] Soderquest K, Hertweck A, Giambartolomei C, Henderson S, Mohamed R, Goldberg R (2017). Genetic variants alter T-bet binding and gene expression in mucosal inflammatory disease. PLoS Genet.

[187] Wu C, Hu Z, Yu D, Huang L, Jin G, Liang J (2009). Genetic variants on chromosome 15q25 associated with lung cancer risk in Chinese populations. Cancer Res United States.

[188] Bernstein DI, Lummus ZL, Kesavalu B, Yao J, Kottyan L, Miller D (2018). Genetic variants with gene regulatory effects are associated with diisocyanate-induced asthma. J Allergy Clin Immunol United States.

[189] Bamji-Mirza M, Li Y, Najem D, Liu QY, Walker D, Lue L-F (2016). Genetic Variations in ABCA7 Can Increase Secreted Levels of Amyloid-β40 and Amyloid-β42 Peptides and ABCA7 Transcription in Cell Culture Models. J Alzheimers Dis Netherlands.

[190] Keller M, Gebhardt C, Huth S, Schleinitz D, Heyne H, Scholz M (2020). Genetically programmed changes in transcription of the novel progranulin regulator. J Mol Med (Berl).

[191] Hou S, Du L, Lei B, Pang CP, Zhang M, Zhuang W (2014). Genome-wide association analysis of Vogt-Koyanagi-Harada syndrome identifies two new susceptibility loci at 1p31.2 and 10q21.3. Nat Genet.

[192] Kawamura R, Tabara Y, Tsukada A, Igase M, Ohashi J, Yamada R (2016). Genome-wide association study of plasma resistin levels identified rs1423096 and rs10401670 as possible functional variants in the Japanese population. Physiol Genomics United States.

[193] Stitzel ML, Sethupathy P, Pearson DS, Chines PS, Song L, Erdos MR (2010). Global epigenomic analysis of primary human pancreatic islets provides insights into type 2 diabetes susceptibility loci. Cell Metab.

[194] Kalita CA, Brown CD, Freiman A, Isherwood J, Wen X, Pique-Regi R (2018). High-throughput characterization of genetic effects on DNA-protein binding and gene transcription. Genome Res.

[195] Zhou Y, Oskolkov N, Shcherbina L, Ratti J, Kock K-H, Su J (2016). HMGB1 binds to the rs7903146 locus in TCF7L2 in human pancreatic islets. Mol Cell Endocrinol Ireland.

[196] Ross-Adams H, Ball S, Lawrenson K, Halim S, Russell R, Wells C (2016). HNF1B variants associate with promoter methylation and regulate gene networks activated in prostate and ovarian cancer. Oncotarget.

[197] Smith EN, D’Antonio-Chronowska A, Greenwald WW, Borja V, Aguiar LR, Pogue R (2019). Human iPSC-derived retinal pigment epithelium: a model system for prioritizing and functionally characterizing causal variants at AMD risk loci. Stem Cell Reports.

[198] Hitomi Y, Kawashima M, Aiba Y, Nishida N, Matsuhashi M, Okazaki H (2015). Human primary biliary cirrhosis-susceptible allele of rs4979462 enhances TNFSF15 expression by binding NF-1. Hum Genet Germany.

[199] López Rodríguez M, Kaminska D, Lappalainen K, Pihlajamäki J, Kaikkonen MU, Laakso M (2017). Identification and characterization of a FOXA2-regulated transcriptional enhancer at a type 2 diabetes intronic locus that controls GCKR expression in liver cells. Genome Med.

[200] Biancolella M, Fortini BK, Tring S, Plummer SJ, Mendoza-Fandino GA, Hartiala J (2014). Identification and characterization of functional risk variants for colorectal cancer mapping to chromosome 11q23.1. Hum Mol Genet.

[201] Flachsbart F, Dose J, Gentschew L, Geismann C, Caliebe A, Knecht C (2017). Identification and characterization of two functional variants in the human longevity gene FOXO3. Nat Commun.

[202] Spracklen CN, Shi J, Vadlamudi S, Wu Y, Zou M, Raulerson CK (2018). Identification and functional analysis of glycemic trait loci in the China Health and Nutrition Survey. PLoS Genet.

[203] Liu L, Pei Y-F, Liu T-L, Hu W-Z, Yang X-L, Li S-C (2019). Identification of a 1p21 independent functional variant for abdominal obesity. Int J Obes (Lond).

[204] Zhou X, Baron RM, Hardin M, Cho MH, Zielinski J, Hawrylkiewicz I (2012). Identification of a chronic obstructive pulmonary disease genetic determinant that regulates HHIP. Hum Mol Genet.

[205] Boulling A, Masson E, Zou W-B, Paliwal S, Wu H, Issarapu P (2017). Identification of a functional enhancer variant within the chronic pancreatitis-associated SPINK1 c.101A>G (p.Asn34Ser)-containing haplotype. Hum Mutat.

[206] Ke J, Tian J, Li J, Gong Y, Yang Y, Zhu Y (2017). Identification of a functional polymorphism affecting microRNA binding in the susceptibility locus 1q25.3 for colorectal cancer. Mol Carcinog.

[207] Alcina A, Fedetz M, Fernández O, Saiz A, Izquierdo G, Lucas M (2013). Identification of a functional variant in the KIF5A-CYP27B1-METTL1-FAM119B locus associated with multiple sclerosis. J Med Genet.

[208] Lo PHY, Urabe Y, Kumar V, Tanikawa C, Koike K, Kato N (2013). Identification of a functional variant in the MICA promoter which regulates MICA expression and increases HCV-related hepatocellular carcinoma risk. PLoS ONE.

[209] Ke J, Lou J, Chen X, Li J, Liu C, Gong Y (2015). Identification of a potential regulatory variant for colorectal cancer risk mapping to chromosome 5q31.1: A Post-GWAS Study. PLoS ONE.

[210] Fogarty MP, Cannon ME, Vadlamudi S, Gaulton KJ, Mohlke KL (2014). Identification of a regulatory variant that binds FOXA1 and FOXA2 at the CDC123/CAMK1D type 2 diabetes GWAS locus. PLoS Genet.

[211] Parker MM, Hao Y, Guo F, Pham B, Chase R, Platig J, et al. Identification of an emphysema-associated genetic variant near TGFB2 with regulatory effects in lung fibroblasts. Elife. 2019;8. 10.7554/eLife.42720 PMC6693893 31343404

[212] Ryoo H, Kong M, Kim Y, Lee C (2013). Identification of functional nucleotide and haplotype variants in the promoter of the CEBPE gene. J Hum Genet England.

[213] van Ouwerkerk AF, Bosada FM, Liu J, Zhang J, van Duijvenboden K, Chaffin M (2020). Identification of functional variant enhancers associated with atrial fibrillation. Circ Res United States.

[214] Castaldi PJ, Guo F, Qiao D, Du F, Naing ZZC, Li Y (2019). Identification of functional variants in the FAM13A chronic obstructive pulmonary disease genome-wide Association Study Locus by Massively Parallel Reporter Assays. Am J Respir Crit Care Med.

[215] Bai W-Y, Wang L, Ying Z-M, Hu B, Xu L, Zhang G-Q (2020). Identification of PIEZO1 polymorphisms for human bone mineral density. Bone.

[216] Fairoozy RH, White J, Palmen J, Kalea AZ, Humphries SE (2016). Identification of the functional variant(s) that explain the low-density lipoprotein receptor (LDLR) GWAS SNP rs6511720 association with lower LDL-C and risk of CHD. PLoS ONE.

[217] Guo X, Lin W, Wen W, Huyghe J, Bien S, Cai Q (2021). Identifying novel susceptibility genes for colorectal cancer risk from a transcriptome-wide association study of 125,478 subjects. Gastroenterology.

[218] Amlie-Wolf A, Tang M, Way J, Dombroski B, Jiang M, Vrettos N (2019). Inferring the molecular mechanisms of noncoding Alzheimer’s disease-associated genetic variants. J Alzheimers Dis.

[219] Hamadou I, Garritano S, Romanel A, Naimi D, Hammada T, Demichelis F. Inherited variant in NFκB-1 promoter is associated with increased risk of IBD in an Algerian population and modulates SOX9 binding. Cancer Rep (Hoboken). 2020;3:e1240. 10.1002/cnr2.1240 PMC7941421 32671985

[220] Pan DZ, Garske KM, Alvarez M, Bhagat YV, Boocock J, Nikkola E (2018). Integration of human adipocyte chromosomal interactions with adipose gene expression prioritizes obesity-related genes from GWAS. Nat Commun.

[221] Zhang X, Cowper-Sal lari R, Bailey SD, Moore JH, Lupien M. Integrative functional genomics identifies an enhancer looping to the SOX9 gene disrupted by the 17q24.3 prostate cancer risk locus. Genome Res. 2012;22:1437–46. 10.1101/gr.135665.111 PMC3409257 22665440

[222] Miller CL, Pjanic M, Wang T, Nguyen T, Cohain A, Lee JD (2016). Integrative functional genomics identifies regulatory mechanisms at coronary artery disease loci. Nat Commun.

[223] Zhang Y, Manjunath M, Zhang S, Chasman D, Roy S, Song JS (2018). Integrative genomic analysis predicts causative Cis-regulatory mechanisms of the Breast Cancer-Associated Genetic Variant rs4415084. Cancer Res.

[224] Berlivet S, Moussette S, Ouimet M, Verlaan DJ, Koka V, Al Tuwaijri A (2012). Interaction between genetic and epigenetic variation defines gene expression patterns at the asthma-associated locus 17q12-q21 in lymphoblastoid cell lines. Hum Genet.

[225] Wang X, Raghavan A, Peters DT, Pashos EE, Rader DJ, Musunuru K (2018). Interrogation of the atherosclerosis-associated SORT1 (Sortilin 1) locus with primary human hepatocytes, induced pluripotent stem cell-hepatocytes, and locus-humanized mice. Arterioscler Thromb Vasc Biol.

[226] Hammaker D, Whitaker JW, Maeshima K, Boyle DL, Ekwall A-KH, Wang W, et al. LBH Gene Transcription Regulation by the Interplay of an Enhancer Risk Allele and DNA Methylation in Rheumatoid Arthritis. Arthritis Rheumatol. 2016;68:2637–45. 10.1002/art.39746 PMC5083131 27159840

[227] Reschen ME, Gaulton KJ, Lin D, Soilleux EJ, Morris AJ, Smyth SS (2015). Lipid-induced epigenomic changes in human macrophages identify a coronary artery disease-associated variant that regulates PPAP2B Expression through Altered C/EBP-beta binding. PLoS Genet.

[228] Zhang Y, Chen X-F, Li J, He F, Li X, Guo Y (2020). lncRNA Neat1 stimulates osteoclastogenesis via sponging miR-7. J Bone Miner Res.

[229] Mei B, Wang Y, Ye W, Huang H, Zhou Q, Chen Y (2019). LncRNA ZBTB40-IT1 modulated by osteoporosis GWAS risk SNPs suppresses osteogenesis. Hum Genet.

[230] Vicente CT, Edwards SL, Hillman KM, Kaufmann S, Mitchell H, Bain L (2015). Long-range modulation of PAG1 expression by 8q21 allergy risk variants. Am J Hum Genet.

[231] Cavalli M, Pan G, Nord H, Wadelius C (2016). Looking beyond GWAS: allele-specific transcription factor binding drives the association of GALNT2 to HDL-C plasma levels. Lipids Health Dis.

[232] Lu X, Zoller EE, Weirauch MT, Wu Z, Namjou B, Williams AH (2015). Lupus risk variant increases pSTAT1 binding and decreases ETS1 expression. Am J Hum Genet.

[233] Choi J, Zhang T, Vu A, Ablain J, Makowski MM, Colli LM (2020). Massively parallel reporter assays of melanoma risk variants identify MX2 as a gene promoting melanoma. Nat Commun.

[234] Elek Z, Németh N, Nagy G, Németh H, Somogyi A, Hosszufalusi N (2015). Micro-RNA binding site polymorphisms in the WFS1 gene are risk factors of diabetes mellitus. PLoS ONE.

[235] Rong H, Gu S, Zhang G, Kang L, Yang M, Zhang J (2017). MiR-2964a-5p binding site SNP regulates ATM expression contributing to age-related cataract risk. Oncotarget.

[236] Elek Z, Dénes R, Prokop S, Somogyi A, Yowanto H, Luo J (2016). Multicapillary gel electrophoresis based analysis of genetic variants in the WFS1 gene. Electrophoresis.

[237] Zhu D-L, Chen X-F, Hu W-X, Dong S-S, Lu B-J, Rong Y (2018). Multiple functional variants at 13q14 risk locus for osteoporosis regulate RANKL expression through long-range super-enhancer. J Bone Miner Res.

[238] He H, Li W, Liyanarachchi S, Srinivas M, Wang Y, Akagi K (2015). Multiple functional variants in long-range enhancer elements contribute to the risk of SNP rs965513 in thyroid cancer. Proc Natl Acad Sci U S A.

[239] Roman TS, Marvelle AF, Fogarty MP, Vadlamudi S, Gonzalez AJ, Buchkovich ML (2015). Multiple hepatic regulatory variants at the GALNT2 GWAS locus associated with high-density lipoprotein cholesterol. Am J Hum Genet.

[240] Bojesen SE, Pooley KA, Johnatty SE, Beesley J, Michailidou K, Tyrer JP, et al. Multiple independent variants at the TERT locus are associated with telomere length and risks of breast and ovarian cancer. Nat Genet. 2013;45:371–84, 384e1–2. 10.1038/ng.2566 PMC3670748 23535731

[241] Beaudoin M, Gupta RM, Won H-H, Lo KS, Do R, Henderson CA (2015). Myocardial infarction-associated SNP at 6p24 interferes with MEF2 binding and associates with PHACTR1 expression levels in human coronary arteries. Arterioscler Thromb Vasc Biol.

[242] John G, Hegarty JP, Yu W, Berg A, Pastor DM, Kelly AA (2011). NKX2-3 variant rs11190140 is associated with IBD and alters binding of NFAT. Mol Genet Metab United States.

[243] Bailey SD, Desai K, Kron KJ, Mazrooei P, Sinnott-Armstrong NA, Treloar AE (2016). Noncoding somatic and inherited single-nucleotide variants converge to promote ESR1 expression in breast cancer. Nat Genet.

[244] Gorbatenko A, Olesen CW, Loebl N, Sigurdsson HH, Bianchi C, Pedraz-Cuesta E (2016). Oncogenic p95HER2 regulates Na+-HCO3- cotransporter NBCn1 mRNA stability in breast cancer cells via 3’UTR-dependent processes. Biochem J England.

[245] Wang Y, Ye W, Liu Y, Mei B, Liu X, Huang Q (2020). Osteoporosis genome-wide association study variant c.3781 C>A is regulated by a novel anti-osteogenic factor miR-345–5p. Hum Mutat.

[246] Zheng J, Huang X, Tan W, Yu D, Du Z, Chang J (2016). Pancreatic cancer risk variant in LINC00673 creates a miR-1231 binding site and interferes with PTPN11 degradation. Nat Genet.

[247] Soldner F, Stelzer Y, Shivalila CS, Abraham BJ, Latourelle JC, Barrasa MI (2016). Parkinson-associated risk variant in distal enhancer of α-synuclein modulates target gene expression. Nature.

[248] Schedel M, Michel S, Gaertner VD, Toncheva AA, Depner M, Binia A (2015). Polymorphisms related to ORMDL3 are associated with asthma susceptibility, alterations in transcriptional regulation of ORMDL3, and changes in TH2 cytokine levels. J Allergy Clin Immunol.

[249] Yang C, Stueve TR, Yan C, Rhie SK, Mullen DJ, Luo J (2018). Positional integration of lung adenocarcinoma susceptibility loci with primary human alveolar epithelial cell epigenomes. Epigenomics.

[250] Oldoni F, Palmen J, Giambartolomei C, Howard P, Drenos F, Plagnol V (2016). Post-GWAS methodologies for localisation of functional non-coding variants: ANGPTL3. Atherosclerosis.

[251] Sakurai D, Zhao J, Deng Y, Kelly JA, Brown EE, Harley JB (2013). Preferential binding to Elk-1 by SLE-associated IL10 risk allele upregulates IL10 expression. PLoS Genet.

[252] Padhy B, Hayat B, Nanda GG, Mohanty PP, Alone DP (2017). Pseudoexfoliation and Alzheimer’s associated CLU risk variant, rs2279590, lies within an enhancer element and regulates CLU, EPHX2 and PTK2B gene expression. Hum Mol Genet.

[253] Bu H, Narisu N, Schlick B, Rainer J, Manke T, Schäfer G (2016). Putative prostate cancer risk SNP in an androgen receptor-binding site of the melanophilin gene illustrates enrichment of risk snps in androgen receptor target sites. Hum Mutat.

[254] Jones SA, Cantsilieris S, Fan H, Cheng Q, Russ BE, Tucker EJ (2019). Rare variants in non-coding regulatory regions of the genome that affect gene expression in systemic lupus erythematosus. Sci Rep.

[255] Richard AC, Peters JE, Savinykh N, Lee JC, Hawley ET, Meylan F (2018). Reduced monocyte and macrophage TNFSF15/TL1A expression is associated with susceptibility to inflammatory bowel disease. PLoS Genet.

[256] Cardinale CJ, March ME, Lin X, Liu Y, Spruce LA, Bradfield JP (2020). Regulation of Janus kinase 2 by an inflammatory bowel disease causal non-coding single nucleotide polymorphism. J Crohns Colitis England.

[257] Qin L, Tiwari AK, Zai CC, Freeman N, Zhai D, Liu F (2020). Regulation of melanocortin-4-receptor (MC4R) expression by SNP rs17066842 is dependent on glucose concentration. Eur Neuropsychopharmacol Netherlands.

[258] Helling BA, Gerber AN, Kadiyala V, Sasse SK, Pedersen BS, Sparks L (2017). Regulation of MUC5B expression in idiopathic pulmonary fibrosis. Am J Respir Cell Mol Biol.

[259] Reinisalo M, Putula J, Mannermaa E, Urtti A, Honkakoski P (2012). Regulation of the human tyrosinase gene in retinal pigment epithelium cells: the significance of transcription factor orthodenticle homeobox 2 and its polymorphic binding site. Mol Vis.

[260] Du M, Zheng R, Ma G, Chu H, Lu J, Li S, et al. Remote modulation of lncRNA GCLET by risk variant at 16p13 underlying genetic susceptibility to gastric cancer. Sci Adv. 2020;6:eaay5525. 10.1126/sciadv.aay5525 PMC7314563 32671202

[261] Pasula S, Tessneer KL, Fu Y, Gopalakrishnan J, Pelikan RC, Kelly JA (2020). Role of systemic lupus erythematosus risk variants with opposing functional effects as a driver of hypomorphic expression of TNIP1 and other genes within a three-dimensional chromatin network. Arthritis Rheumatol.

[262] Yang Y-C, Fu W-P, Zhang J, Zhong L, Cai S-X, Sun C (2018). rs401681 and rs402710 confer lung cancer susceptibility by regulating TERT expression instead of CLPTM1L in East Asian populations. Carcinogenesis England.

[263] Pan G, Cavalli M, Carlsson B, Skrtic S, Kumar C, Wadelius C. rs953413 Regulates polyunsaturated fatty acid metabolism by modulating ELOVL2 expression. iScience. 2020;23:100808. 10.1016/j.isci.2019.100808 PMC7033636 31928966

[264] Nanda GG, Kumar MV, Pradhan L, Padhy B, Sundaray S, Das S (2018). rs4246215 is targeted by hsa-miR1236 to regulate FEN1 expression but is not associated with Fuchs’ endothelial corneal dystrophy. PLoS ONE.

[265] Hauberg ME, Holm-Nielsen MH, Mattheisen M, Askou AL, Grove J, Børglum AD (2016). Schizophrenia risk variants affecting microRNA function and site-specific regulation of NT5C2 by miR-206. Eur Neuropsychopharmacol.

[266] Hou Y, Liang W, Zhang J, Li Q, Ou H, Wang Z (2018). Schizophrenia-associated rs4702 G allele-specific downregulation of FURIN expression by miR-338-3p reduces BDNF production. Schizophr Res.

[267] Guillen-Guio B, Lorenzo-Salazar JM, Ma S-F, Hou P-C, Hernandez-Beeftink T, Corrales A (2020). Sepsis-associated acute respiratory distress syndrome in individuals of European ancestry: a genome-wide association study. Lancet Respir Med.

[268] Xiao F, Zhang P, Wang Y, Tian Y, James M, Huang C-C (2020). Single-nucleotide polymorphism rs13426236 contributes to an increased prostate cancer risk via regulating MLPH splicing variant 4. Mol Carcinog.

[269] Hou G, Harley ITW, Lu X, Zhou T, Xu N, Yao C (2021). SLE non-coding genetic risk variant determines the epigenetic dysfunction of an immune cell specific enhancer that controls disease-critical microRNA expression. Nat Commun.

[270] Fortini BK, Tring S, Devall MA, Ali MW, Plummer SJ, Casey G (2021). SNPs associated with colorectal cancer at 15q13.3 affect risk enhancers that modulate GREM1 gene expression. Hum Mutat.

[271] Liu S, Liu Y, Zhang Q, Wu J, Liang J, Yu S (2017). Systematic identification of regulatory variants associated with cancer risk. Genome Biol.

[272] Kong X, Sawalha AH (2019). Takayasu arteritis risk locus in IL6 represses the anti-inflammatory gene GPNMB through chromatin looping and recruiting MEF2-HDAC complex. Ann Rheum Dis.

[273] Wang S, Wen F, Tessneer KL, Gaffney PM (2016). TALEN-mediated enhancer knockout influences TNFAIP3 gene expression and mimics a molecular phenotype associated with systemic lupus erythematosus. Genes Immun.

[274] Wei R, Cao L, Pu H, Wang H, Zheng Y, Niu X (2015). TERT Polymorphism rs2736100-C is associated with EGFR mutation-positive non-small cell lung cancer. Clin Cancer Res.

[275] Sheng X, Tong N, Tao G, Luo D, Wang M, Fang Y (2013). TERT polymorphisms modify the risk of acute lymphoblastic leukemia in Chinese children. Carcinogenesis England.

[276] Lubbe SJ, Pittman AM, Olver B, Lloyd A, Vijayakrishnan J, Naranjo S (2012). The 14q22.2 colorectal cancer variant rs4444235 shows cis-acting regulation of BMP4. Oncogene.

[277] Ghanbari M, Sedaghat S, de Looper HWJ, Hofman A, Erkeland SJ, Franco OH, et al. The association of common polymorphisms in miR-196a2 with waist to hip ratio and miR-1908 with serum lipid and glucose. Obesity (Silver Spring); 2015;23:495–503. 10.1002/oby.20975 25557604

[278] Prestel M, Prell-Schicker C, Webb T, Malik R, Lindner B, Ziesch N (2019). The atherosclerosis risk variant rs2107595 mediates allele-specific transcriptional regulation of HDAC9 via E2F3 and Rb1. Stroke United States.

[279] Tuupanen S, Turunen M, Lehtonen R, Hallikas O, Vanharanta S, Kivioja T (2009). The common colorectal cancer predisposition SNP rs6983267 at chromosome 8q24 confers potential to enhanced Wnt signaling. Nat Genet.

[280] Matthews SM, Eshelman MA, Berg AS, Koltun WA, Yochum GS (2019). The Crohn’s disease associated SNP rs6651252 impacts MYC gene expression in human colonic epithelial cells. PLoS ONE.

[281] Li D, Zhu G, Lou S, Ma L, Zhang C, Pan Y (2020). The functional variant of NTN1 contributes to the risk of nonsyndromic cleft lip with or without cleft palate. Eur J Hum Genet.

[282] Vecellio M, Roberts AR, Cohen CJ, Cortes A, Knight JC, Bowness P (2016). The genetic association of RUNX3 with ankylosing spondylitis can be explained by allele-specific effects on IRF4 recruitment that alter gene expression. Ann Rheum Dis.

[283] Deng Y, Li P, Liu W, Pu R, Yang F, Song J (2020). The genetic polymorphism down-regulating HLA-DRB1 enhancer activity facilitates HBV persistence, evolution and hepatocarcinogenesis in the Chinese Han population. J Viral Hepat England.

[284] Yang S, Gao Y, Liu G, Li J, Shi K, Du B (2015). The human ATF1 rs11169571 polymorphism increases essential hypertension risk through modifying miRNA binding. FEBS Lett England.

[285] Li C, Yu Q, Han L, Wang C, Chu N, Liu S (2014). The hURAT1 rs559946 polymorphism and the incidence of gout in Han Chinese men. Scand J Rheumatol.

[286] Wang L, Li H, Yang B, Guo L, Han X, Li L (2017). The hypertension risk variant Rs820430 functions as an enhancer of SLC4A7. Am J Hypertens.

[287] Syddall CM, Reynard LN, Young DA, Loughlin J (2013). The identification of trans-acting factors that regulate the expression of GDF5 via the osteoarthritis susceptibility SNP rs143383. PLoS Genet.

[288] Zhou L, Fu G, Wei J, Shi J, Pan W, Ren Y (2016). The identification of two regulatory ESCC susceptibility genetic variants in the TERT-CLPTM1L loci. Oncotarget.

[289] Shao L, Zuo X, Yang Y, Zhang Y, Yang N, Shen B (2019). The inherited variations of a p53-responsive enhancer in 13q12.12 confer lung cancer risk by attenuating TNFRSF19 expression. Genome Biol.

[290] Tuo XM, Zhu DL, Chen XF, Rong Y, Guo Y, Yang TL (2020). The osteoporosis susceptible SNP rs4325274 remotely regulates the SOX6 gene through enhancers. Yi Chuan China.

[291] Richardson K, Louie-Gao Q, Arnett DK, Parnell LD, Lai C-Q, Davalos A (2011). The PLIN4 variant rs8887 modulates obesity related phenotypes in humans through creation of a novel miR-522 seed site. PLoS ONE.

[292] Jendrzejewski J, He H, Radomska HS, Li W, Tomsic J, Liyanarachchi S (2012). The polymorphism rs944289 predisposes to papillary thyroid carcinoma through a large intergenic noncoding RNA gene of tumor suppressor type. Proc Natl Acad Sci USA.

[293] Kong HK, Yoon S, Park JH (2012). The regulatory mechanism of the LY6K gene expression in human breast cancer cells. J Biol Chem.

[294] Wang Y, He H, Liyanarachchi S, Genutis LK, Li W, Yu L (2018). The role of SMAD3 in the genetic predisposition to papillary thyroid carcinoma. Genet Med.

[295] Afanasyeva MA, Putlyaeva LV, Demin DE, Kulakovskiy IV, Vorontsov IE, Fridman MV (2017). The single nucleotide variant rs12722489 determines differential estrogen receptor binding and enhancer properties of an IL2RA intronic region. PLoS ONE.

[296] Xia Q, Chesi A, Manduchi E, Johnston BT, Lu S, Leonard ME (2016). The type 2 diabetes presumed causal variant within TCF7L2 resides in an element that controls the expression of ACSL5. Diabetologia Germany.

[297] Mellado-Gil JM, Fuente-Martín E, Lorenzo PI, Cobo-Vuilleumier N, López-Noriega L, Martín-Montalvo A (2018). The type 2 diabetes-associated HMG20A gene is mandatory for islet beta cell functional maturity. Cell Death Dis.

[298] Kamens HM, Miyamoto J, Powers MS, Ro K, Soto M, Cox R (2015). The β3 subunit of the nicotinic acetylcholine receptor: modulation of gene expression and nicotine consumption. Neuropharmacology.

[299] Pattison JM, Posternak V, Cole MD (2016). Transcription factor KLF5 binds a cyclin E1 polymorphic intronic enhancer to confer increased bladder cancer risk. Mol Cancer Res.

[300] Ding C, Zhang C, Kopp R, Kuney L, Meng Q, Wang L, et al. Transcription factor POU3F2 regulates TRIM8 expression contributing to cellular functions implicated in schizophrenia. Mol Psychiatry. 2020; 10.1038/s41380-020-00877-2 PMC7956165 32929213

[301] Liu W, Anstee QM, Wang X, Gawrieh S, Gamazon ER, Athinarayanan S (2016). Transcriptional regulation of PNPLA3 and its impact on susceptibility to nonalcoholic fatty liver Disease (NAFLD) in humans. Aging (Albany NY).

[302] Guthridge JM, Lu R, Sun H, Sun C, Wiley GB, Dominguez N (2014). Two functional lupus-associated BLK promoter variants control cell-type- and developmental-stage-specific transcription. Am J Hum Genet.

[303] Liu L, Yang X-L, Zhang H, Zhang Z-J, Wei X-T, Feng G-J (2020). Two novel pleiotropic loci associated with osteoporosis and abdominal obesity. Hum Genet.

[304] Lewis MJ, Vyse S, Shields AM, Boeltz S, Gordon PA, Spector TD (2015). UBE2L3 polymorphism amplifies NF-κB activation and promotes plasma cell development, linking linear ubiquitination to multiple autoimmune diseases. Am J Hum Genet.

[305] Dryden NH, Broome LR, Dudbridge F, Johnson N, Orr N, Schoenfelder S (2014). Unbiased analysis of potential targets of breast cancer susceptibility loci by Capture Hi-C. Genome Res.

[306] Wright JB, Brown SJ, Cole MD (2010). Upregulation of c-MYC in cis through a large chromatin loop linked to a cancer risk-associated single-nucleotide polymorphism in colorectal cancer cells. Mol Cell Biol.

[307] Smith AJP, Howard P, Shah S, Eriksson P, Stender S, Giambartolomei C (2012). Use of allele-specific FAIRE to determine functional regulatory polymorphism using large-scale genotyping arrays. PLoS Genet.

[308] Wang X, Hayes JE, Xu X, Gao X, Mehta D, Lilja HG (2021). Validation of prostate cancer risk variants rs10993994 and rs7098889 by CRISPR/Cas9 mediated genome editing. Gene.

[309] Sribudiani Y, Metzger M, Osinga J, Rey A, Burns AJ, Thapar N (2011). Variants in RET associated with Hirschsprung’s disease affect binding of transcription factors and gene expression. Gastroenterology.

[310] Vincentz JW, Firulli BA, Toolan KP, Arking DE, Sotoodehnia N, Wan J (2019). Variation in a left ventricle-specific Hand1 enhancer impairs GATA transcription factor binding and disrupts conduction system development and function. Circ Res.

[311] Shirts BH, Howard MT, Hasstedt SJ, Nanjee MN, Knight S, Carlquist JF (2012). Vitamin D dependent effects of APOA5 polymorphisms on HDL cholesterol. Atherosclerosis.

[312] Chen G, Ribeiro CMP, Sun L, Okuda K, Kato T, Gilmore RC (2019). XBP1S regulates MUC5B in a promoter variant-dependent pathway in idiopathic pulmonary fibrosis airway epithelia. Am J Respir Crit Care Med.

[313] Mizuta I, Takafuji K, Ando Y, Satake W, Kanagawa M, Kobayashi K (2013). YY1 binds to α-synuclein 3’-flanking region SNP and stimulates antisense noncoding RNA expression. J Hum Genet England.

[314] Cano-Gamez E, Trynka G. From GWAS to Function: Using Functional Genomics to Identify the Mechanisms Underlying Complex Diseases. Front Genet [Internet]. 2020 [cited 2020 Jun 8];11. Available from: https://www.ncbi.nlm.nih.gov/pmc/articles/PMC7237642/10.3389/fgene.2020.00424 PMC7237642 32477401

[315] Edwards SL, Beesley J, French JD, Dunning AM (2013). Beyond GWASs: illuminating the dark road from association to function. Am J Human Genet.

[316] Bulik-Sullivan BK, Loh P-R, Finucane H, Ripke S, Yang J, Patterson N (2015). LD score regression distinguishes confounding from polygenicity in genome-wide association studies. Nat Genet.

[317] Heritability of >4,000 traits & disorders in UK Biobank [Internet]. [cited 2022 Feb 4]. Available from: https://nealelab.github.io/UKBB_ldsc/

[318] Perenthaler E, Yousefi S, Niggl E, Barakat TS. Beyond the Exome: The Non-coding Genome and Enhancers in Neurodevelopmental Disorders and Malformations of Cortical Development. Front Cell Neurosci [Internet]. Frontiers; 2019 [cited 2021 Jun 10];13. Available from: 10.3389/fncel.2019.00352/full 10.3389/fncel.2019.00352 PMC6685065 31417368

[319] French JD, Edwards SL (2020). The role of noncoding variants in heritable disease. Trends Genet.

[320] Rojano E, Seoane P, Ranea JAG, Perkins JR (2019). Regulatory variants: from detection to predicting impact. Brief Bioinform.

[321] Moore LD, Le T, Fan G (2013). DNA methylation and its basic function. Neuropsychopharmacology.

[322] Lowdon RF, Jang HS, Wang T (2016). Evolution of epigenetic regulation in vertebrate genomes. Trends Genet.

[323] Zhang P, Wu W, Chen Q, Chen M. Non-Coding RNAs and their Integrated Networks. J Integr Bioinform [Internet]. 2019 [cited 2021 May 31];16. Available from: https://www.ncbi.nlm.nih.gov/pmc/articles/PMC6798851/10.1515/jib-2019-0027 PMC6798851 31301674

[324] Cammaerts S, Strazisar M, De Rijk P, Del Favero J (2015). Genetic variants in microRNA genes: impact on microRNA expression, function, and disease. Front Genet.

[325] Felekkis K, Touvana E, Stefanou C, Deltas C (2010). microRNAs: a newly described class of encoded molecules that play a role in health and disease. Hippokratia.

[326] Steri M, Idda ML, Whalen MB, Orrù V (2018). Genetic variants in mRNA untranslated regions. Wiley Interdiscip Rev RNA..

[327] A M, M G, Jf C, R B. SNPs in microRNA target sites and their potential role in human disease. Open biology [Internet]. Open Biol; 2017 [cited 2021 May 31];7. Available from: https://pubmed.ncbi.nlm.nih.gov/28381629/10.1098/rsob.170019 PMC5413909 28381629

[328] Statello L, Guo C-J, Chen L-L, Huarte M (2021). Gene regulation by long non-coding RNAs and its biological functions. Nature Rev Mol Cell Biol..

[329] Giral H, Landmesser U, Kratzer A. Into the Wild: GWAS Exploration of Non-coding RNAs. Front Cardiovasc Med [Internet]. Frontiers; 2018 [cited 2021 Jun 10];5. Available from: 10.3389/fcvm.2018.00181/full 10.3389/fcvm.2018.00181 PMC6304420 30619888

[330] Gasperini M, Hill AJ, McFaline-Figueroa JL, Martin B, Kim S, Zhang MD (2019). A genome-wide framework for mapping gene regulation via cellular genetic screens. Cell.

[331] Schraivogel D, Gschwind AR, Milbank JH, Leonce DR, Jakob P, Mathur L, et al. Targeted Perturb-seq enables genome-scale genetic screens in single cells. Nat Methods [Internet]. 2020 [cited 2020 Jun 2]; Available from: http://www.nature.com/articles/s41592-020-0837-5 10.1038/s41592-020-0837-5 PMC7610614 32483332

[332] Boix CA, James BT, Park YP, Meuleman W, Kellis M (2021). Regulatory genomic circuitry of human disease loci by integrative epigenomics. Nature.

[333] Doni Jayavelu N, Jajodia A, Mishra A, Hawkins RD. Candidate silencer elements for the human and mouse genomes. Nat Commun [Internet]. 2020 [cited 2020 Apr 21];11. Available from: https://www.ncbi.nlm.nih.gov/pmc/articles/PMC7044160/10.1038/s41467-020-14853-5 PMC7044160 32103011

[334] Gasperini M, Tome JM, Shendure J (2020). Towards a comprehensive catalogue of validated and target-linked human enhancers. Nat Rev Genet.

[335] Pang B, Snyder MP (2020). Systematic identification of silencers in human cells. Nat Genet.

[336] Bramer WM, de Jonge GB, Rethlefsen ML, Mast F, Kleijnen J (2018). A systematic approach to searching: an efficient and complete method to develop literature searches. J Med Libr Assoc.

[337] Bramer WM, Giustini D, Kramer BMR (2016). Comparing the coverage, recall, and precision of searches for 120 systematic reviews in Embase, MEDLINE, and Google Scholar: a prospective study. Syst Rev.

[338] Wang X, Tucker NR, Rizki G, Mills R, Krijger PH, de Wit E, et al. Discovery and validation of sub-threshold genome-wide association study loci using epigenomic signatures. Elife. 2016;5. 10.7554/eLife.10557 PMC4862755 27162171

[339] Stadhouders R, Aktuna S, Thongjuea S, Aghajanirefah A, Pourfarzad F, van Ijcken W (2014). HBS1L-MYB intergenic variants modulate fetal hemoglobin via long-range MYB enhancers. J Clin Invest.

[340] Pashos EE, Park Y, Wang X, Raghavan A, Yang W, Abbey D (2017). Large, diverse population cohorts of hiPSCs and derived hepatocyte-like cells reveal functional genetic variation at blood lipid-associated loci. Cell Stem Cell.

[341] Visser M, Kayser M, Palstra R-J (2012). HERC2 rs12913832 modulates human pigmentation by attenuating chromatin-loop formation between a long-range enhancer and the OCA2 promoter. Genome Res.

[342] Musunuru K, Strong A, Frank-Kamenetsky M, Lee NE, Ahfeldt T, Sachs KV (2010). From noncoding variant to phenotype via SORT1 at the 1p13 cholesterol locus. Nature.

[343] Guo H, Ahmed M, Zhang F, Yao CQ, Li S, Liang Y (2016). Modulation of long noncoding RNAs by risk SNPs underlying genetic predispositions to prostate cancer. Nat Genet.

[344] Ghoussaini M, French JD, Michailidou K, Nord S, Beesley J, Canisus S (2016). Evidence that the 5p12 variant rs10941679 confers susceptibility to estrogen-receptor-positive breast cancer through FGF10 and MRPS30 regulation. Am J Hum Genet.

[345] Viñuela A, Varshney A, van de Bunt M, Prasad RB, Asplund O, Bennett A (2020). Genetic variant effects on gene expression in human pancreatic islets and their implications for T2D. Nat Commun.

[346] Stacey D, Fauman EB, Ziemek D, Sun BB, Harshfield EL, Wood AM (2019). ProGeM: a framework for the prioritization of candidate causal genes at molecular quantitative trait loci. Nucleic Acids Res.

[347] Fang H, ULTRA-DD Consortium, De Wolf H, Knezevic B, Burnham KL, Osgood J, et al. A genetics-led approach defines the drug target landscape of 30 immune-related traits. Nat Genet. 2019;51:1082–91. 10.1038/s41588-019-0456-1 PMC7124888 31253980

[348] Lukowski SW, Lloyd-Jones LR, Holloway A, Kirsten H, Hemani G, Yang J (2017). Genetic correlations reveal the shared genetic architecture of transcription in human peripheral blood. Nat Commun.

