# Supplementary material for: The landscape of GWAS validation; systematic review identifying 309 validated non-coding variants across 130 human diseases
Source: BMC Med Genomics. 2022 Apr 1;15:74. doi: 10.1186/s12920-022-01216-w (PMC8973751; doi:10.1186/s12920-022-01216-w)
Supplement: Supplementary file 1 — Additional file 1 contains exact search terms and criteria used for creating the initial broad literature search. [file 12920_2022_1216_MOESM1_ESM.docx]

**Broad Systematic Literature Search criteria and terms.**

| **Sub-Search** | **Search Strategy using Search Concepts as defined below** | **Comment** | **#Unique PMIDs in broad Search** | **#Articles in final set (after GWAS catalog cross-referencing)** |
| --- | --- | --- | --- | --- |
| **A** | (Variant OR SNP OR rsID OR Allele-specific) AND ("Non-coding") AND ("Functional Variant" OR "Regulatory Variant" OR "Causal Variant" OR "Functional Other") | Non-coding explicitly mentioned, no GWAS constraint | 3072 | 75 |
| **B** | (Variant OR SNP OR rsID OR Allele-specific) AND ("Non-Coding Context") AND ("Functional Variant" OR "Regulatory Variant" OR "Causal Variant" OR "Functional Other") | Sub Search A but “non-coding context” instead of noncoding explicitly mentioned; non-coding context: terms/concepts describing non-coding regions of the genome (enhancer, silencer, insulator, promoter, UTR, untranslated region, intron, regulatory element) OR the mechanism by which variant functions (terms included transcription factor (binding), chromatin, histone, CTCF, cis-regulation) OR miRNA | 26041 | 271 |
| **C** | (rsID) AND (GWAS OR Genetic Association Studies) AND ("Non-coding" OR "Non-Coding Context") | GWAS + rsId + non-coding (explicitely/context) | 5442 | 184 |
| **D** | (Variant OR SNP OR rsID OR Alleles OR Polymorphism OR Locus OR Allele-specific) AND (GWAS OR Genetic Association Studies) AND ("Functional Variant/SNP" OR "Regulatory Variant/SNP" OR "Causal Variant/SNP" OR Functional) | GWAS + functional (variant) / regulatory variant / causal variant | 10434 | 152 |
| **Total (A+B+C+D)** |  |  | **36676** | **286** |

| **Used in Sub Search** | **Search Concept** [referenced as concept: in 'Terms / Ontology classes'] | **Terms / Ontology classes** [explanation: N/n : two search terms, in any order, within a specified number of n words apart (max WordGap = n).; P/n: one search term appears within a specified number of n words before a second term, search term 1 preceeds search term 2; S/n: two search terms appear in the same sentence, any order, no maximum WordGap] | **Settings** | **Ontology Source** |
| --- | --- | --- | --- | --- |
| A; B; D | Variant | variant | {text:variant, morphoVariants: true} | n/a |
| A; B; D | SNP | "single nucleotide polymorphism" OR "single-nucleotide polymorphisms" OR "single nucleotide polymorphisms" OR "single-nucleotide variation" OR "single nucleotide variation" OR "single-nucleotide variant" OR "single nucleotide variant”, class: {snid: nlm.D020641} | As Macro | nlm->MESH 2021 |
| A; B; C; D | rsID | class: {snid: mutation.rsid, pt: rsID} | default | Linguamatics I2E |
| A; B; D | Allele-specific | allele-specific OR allele N/0 specific | default | n/a |
| A; B; D | Locus | class: {snid: nlm.D056426, pt: Genetic Loci} OR class: {snid: nci.C45822, pt: Locus} | default | nlm->MESH 2021, nci-> NCI Derived 20.07d |
| A; B; D | Alleles | class: {snid: nlm.D000483, pt: Alleles} | default | nlm->MESH 2021 |
| A; B; D | Polymorphism | Polymorphism OR polymorphisms | default | n/a |
| A; C | Non-Coding | “non-coding” OR non P/0 coding OR noncoding | default | n/a |
| A; B | Causal Variant | (causal OR causative) P/5 (concept:Variant OR concept:SNP OR concept:rsID OR concept:Locus OR concept:Alleles OR concept:Polymorphism OR gene) | {text: gene, morphoVariants: true} | n/a |
| A; B | Functional Variant | functional P/5 (concept:Variant OR concept:SNP OR concept:rsID OR concept:Allele-specific OR concept:Locus OR Alleles OR concept:Polymorphism OR gene) | {text: gene, morphoVariants: true} | n/a |
| A; B | Regulatory Variant | regulatory P/5 (concept:Variant OR concept:SNP OR concept:rsID OR concept:Allele-specific OR concept:Locus OR concept:Alleles OR concept:Polymorphism OR gene) | {text: gene, morphoVariants: true} | n/a |
| D | Causal Variant/SNP | (causal OR causative) P/1 (concept:Variant OR concept:SNP) | default | n/a |
| D | Functional Variant/SNP | functional P/1 (concept:Variant OR concept:SNP) | default | n/a |
| D | Regulatory Variant/SNP | regulatory P/1 (concept:Variant OR concept:SNP) | default | n/a |
| A; B; D | Functional | functional OR functionally | default |  |
| A; B | Functional Other | (causal OR concept:Functional OR Function OR Mechanism OR Mechanistical OR Mechanistically OR causative OR causality OR role OR effect) S/0 (concept:SNP OR concept:rsID OR concept:Allele-specific OR concept:Locus OR concept:Alleles OR concept:Polymorphism OR gene OR concept:GWAS) | {text: function, morphoVariants: true}, {text: mechanism, morphoVariants: true}, {text: effect, morphoVariants: true}, {text: gene, morphoVariants: true} | n/a |
| C;D | GWAS | GWAS OR "GWA studies" OR "GWA study" OR "genome wide association study" OR "genome wide association studies" OR "genome wide association analysis” OR "genome wide association analyses" OR "whole genome association study" OR WGAS OR “WGA studies” OR “WGA study” OR class: {snid: nci.C93020} | As Macro | nci-> NCI Derived 20.07d |
| C;D | Genetic Association Studies | class: {snid: nlm.D056726, pt: Genetic Association Studies} | default | nlm->MESH 2021 |
| B; C | Non-Coding Context | concept:miRNA OR concept:Enhancer OR concept:Silencer OR concept:Promoter OR concept:Insulator OR concept:"Untranslated Region" OR concept:"Regulatory Element" OR concept:"Transcription Factor Binding" OR concept:"Transcription Factor" OR concept:Intron OR concept:Cis-Regulation OR concept:Chromatin OR concept:Histone OR concept:CTCF OR concept:Intragenic OR concept:Intergenic OR concept:Intronic | default | n/a |
| B; C | miRNA | "mir\d+": {matchType: Regexp} OR "mir-\d+": {matchType: Regexp} OR "mir\d+": {matchType: Regexp} OR class: {snid: nlm.D035683, pt: MicroRNAs | default | ncbi -> Entrez Gene, nlm->MESH 2021, |
| B; C | Enhancer | enhancer [optional: P/2 (enhancer OR sequence OR activity OR binding OR region)] | {text: element, morphoVariants: true}, {text: sequence, morphoVariants: true}, {text: region, morphoVariants: true} | n/a |
| B; C | Silencer | silencer [optional: P/2 (enhancer OR sequence OR activity OR binding OR region)] | {text: element, morphoVariants: true}, {text: sequence, morphoVariants: true}, {text: region, morphoVariants: true} | n/a |
| B; C | Promoter | promoter [optional: P/2 (enhancer OR sequence OR activity OR binding OR region)] | {text: element, morphoVariants: true}, {text: sequence, morphoVariants: true}, {text: region, morphoVariants: true} | n/a |
| B; C | Insulator | insulator [optional: P/2 (enhancer OR sequence OR activity OR binding OR region)] | {text: element, morphoVariants: true}, {text: sequence, morphoVariants: true}, {text: region, morphoVariants: true} | n/a |
| B; C | Untranslated Region | (untranslated P/0 region) OR UTR | {text: region, morphoVariants: true}, {text: UTR, caseSensitive: true} | n/a |
| B; C | Regulatory Element | regulatory P/3 (enhancer OR sequence OR activity OR binding OR region OR DNA) | {text: element, morphoVariants: true}, {text: sequence, morphoVariants: true}, {text: region, morphoVariants: true}, {text: DNA, caseSensitive: true} | n/a |
| B; C | Transcription Factor Binding | TF-binding OR (("transcription factor" OR transcription-factor OR TF) N/2 (bind*: {matchType: Wildcard})) | {text: TF, caseSensitive: true} | n/a |
| B; C | Transcription Factor | transcription factor: {matchType: Substring} OR transcription-factor: {matchType: Substring} | default | n/a |
| B; C | Cis-Regulation | ((cis OR "cis-":{matchType: Substring}) P/0 "regulat": {matchType: Substring}) OR "cis-regulat": {matchType: Substring} | default | n/a |
| B; C | Chromatin | chromatin: {matchType: Substring} | default | n/a |
| B; C | Histone | histone: {matchType: Substring} | default | n/a |
| B; C | CTCF | CTCF: {matchType: Substring} | {text: CTCF, caseSensitive: true} | n/a |
| B; C | Intergenic | intergenic | default | n/a |
| B; C | Intragenic | intragenic | default | n/a |
| B; C | Intron | intron | {text: intron, morphoVariants: true} | n/a |
| B; C | Intronic | intronic | default | n/a |
