# Supplementary material for: The landscape of GWAS validation; systematic review identifying 309 validated non-coding variants across 130 human diseases
Source: BMC Med Genomics. 2022 Apr 1;15:74. doi: 10.1186/s12920-022-01216-w (PMC8973751; doi:10.1186/s12920-022-01216-w)
Supplement: Supplementary file 2 — Additional file 2 contains exact terms and phrases used to setup the seven filters that were used to narrow down the broad search results. [file 12920_2022_1216_MOESM2_ESM.docx]

**Natural language processing and text-mining filters criteria and terms**

| **Filter Category** | **Filter Concept** | **Terms / Ontology classes** [explanation: N/n : two search terms, in any order, within a specified number of words n apart (max WordGap = n).; P/n: one search term appears within a specified number of words n before a second term, search term 1 preceeds search term 2; S/n: two search terms appear in the same sentence, any order, no maximum WordGap] | **Settings** | **Ontology Source** | **Tool** |
| --- | --- | --- | --- | --- | --- |
| Genes / Proteins | human protein coding genes | GENE vocabulary covers all human genes from HGNC standard list (enriched synonym lists) | subsume': 'true', 'rejectMinorHits' : 'true', 'fuzzy' : 'true' + plus internal disambiguation workflow | enriched HGNC, Entrez Gene and Uniprot ontologies | SciBite TERMite |
| miRNA | list of microRNAs | MIRNA vocabulary | subsume': 'true', 'rejectMinorHits' : 'true', 'fuzzy' : 'true' + plus internal disambiguation workflow | algorithm | SciBite TERMite |
| Diseases | list of human diseases | INDICATION vocabulary covers diseases from MESH C-branch ("Diseases") and MESH-F ("Psychiatry and Psychology") branch plus additional diseases (enriched synonym lists) | subsume': 'true', 'rejectMinorHits' : 'true', 'fuzzy' : 'true' + plus internal disambiguation workflow | MESH enriched & expanded | SciBite TERMite |
| Publication Type | Research Article | Publication Type "Journal Article" NOT "Review" | default | MESH | n/a |
| Non-coding Context | Enhancer | enhancer [optional: P/2 (enhancer OR sequence OR activity OR binding OR region)] | {text: element, morphoVariants: true}, {text: sequence, morphoVariants: true}, {text: region, morphoVariants: true} | n/a | Linguamatics I2E |
| Non-coding Context | Insulator | insulator [optional: P/2 (enhancer OR sequence OR activity OR binding OR region)] | {text: element, morphoVariants: true}, {text: sequence, morphoVariants: true}, {text: region, morphoVariants: true} | n/a | Linguamatics I2E |
| Non-coding Context | Intergenic | intergenic | default | n/a | Linguamatics I2E |
| Non-coding Context | Intragenic | intragenic | default | n/a | Linguamatics I2E |
| Non-coding Context | Intron | intron | {text: intron, morphoVariants: true} | n/a | Linguamatics I2E |
| Non-coding Context | Intronic | intronic | default | n/a | Linguamatics I2E |
| Non-coding Context | Non-Coding | “non-coding” OR non P/0 coding OR noncoding | default | n/a | Linguamatics I2E |
| Non-coding Context | miRNA | "mir\d+": {matchType: Regexp} OR "mir-\d+": {matchType: Regexp} OR "mir\d+": {matchType: Regexp} OR class: {snid: nlm.D035683, pt: MicroRNAs} | default | ncbi -> Entrez Gene, nlm->MESH 2021, | Linguamatics I2E |
| Non-coding Context | Promoter | promoter [optional: P/2 (enhancer OR sequence OR activity OR binding OR region)] | {text: element, morphoVariants: true}, {text: sequence, morphoVariants: true}, {text: region, morphoVariants: true} | n/a | Linguamatics I2E |
| Non-coding Context | Regulatory Element | regulatory P/3 (enhancer OR sequence OR activity OR binding OR region OR DNA) | {text: element, morphoVariants: true}, {text: sequence, morphoVariants: true}, {text: region, morphoVariants: true}, {text: DNA, caseSensitive: true} | n/a | Linguamatics I2E |
| Non-coding Context | Silencer | silencer [optional: P/2 (enhancer OR sequence OR activity OR binding OR region)] | {text: element, morphoVariants: true}, {text: sequence, morphoVariants: true}, {text: region, morphoVariants: true} | n/a | Linguamatics I2E |
| Non-coding Context | Untranslated Region | (untranslated P/0 region) OR UTR | {text: region, morphoVariants: true}, {text: UTR, caseSensitive: true} | n/a | Linguamatics I2E |
| Genetic Association | Association | association: {matchType: Substring} OR associated: {matchType: Substring} | default | n/a | Linguamatics I2E |
| Genetic Association | Genetic Association Studies | class: {snid: nlm.D056726, pt: Genetic Association Studies} | default | nlm->MESH 2021 | Linguamatics I2E |
| Genetic Association | GWAS | GWAS OR "GWA studies" OR "GWA study" OR "genome wide association study" OR "genome-wide association study" OR "genome wide association studies" OR "genome-wide association studies" OR "genome wide association analysis" OR "genome-wide association analysis" OR "genome wide association analyses" OR "genome-wide association analyses" OR "whole genome association study" OR "whole genome association studies” OR WGAS OR “WGA studies” OR “WGA study” OR class: {snid: nci.C93020} | As Macro | nci-> NCI Derived 20.07d | Linguamatics I2E |
| Genetic Association | Locus | class: {snid: nlm.D056426, pt: Genetic Loci} OR class: {snid: nci.C45822, pt: Locus} | default | nlm->MESH 2021, nci-> NCI Derived 20.07d | Linguamatics I2E |
| Functional Analysis | rsID | class: {snid: mutation.rsid, pt: rsID} | default | Linguamatics I2E | Linguamatics I2E |
| Functional Analysis | Causal Variant | (causal OR causative) P/5 (concept:Variant OR concept:SNP OR concept:rsID OR concept:Locus OR concept:Alleles OR concept:Polymorphism OR gene) | {text: gene, morphoVariants: true} | n/a | Linguamatics I2E |
| Functional Analysis | Functional Variant | functional P/5 (concept:Variant OR concept:SNP OR concept:rsID OR concept:Allele-specific OR concept:Locus OR Alleles OR concept:Polymorphism OR gene) | {text: gene, morphoVariants: true} | n/a | Linguamatics I2E |
| Functional Analysis | Regulatory Variant | regulatory P/5 (concept:Variant OR concept:SNP OR concept:rsID OR concept:Allele-specific OR concept:Locus OR concept:Alleles OR concept:Polymorphism OR gene) | {text: gene, morphoVariants: true} | n/a | Linguamatics I2E |
| Experimental Validation | Allele-specific | allele-specific OR allele N/0 specific | default | n/a | Linguamatics I2E |
| Experimental Validation | Luciferase | Luciferase: {matchType: Substring} | {text: Luciferase, matchType: Substring} | n/a | Linguamatics I2E |
| Experimental Validation | Luciferase Assay | dual-luciferase OR luciferase-assay OR (dual N/2 Luciferase: {matchType: Substring} N/2 Assay:{matchType: Substring} | {text: Luciferase, morphoVariants: true}, {text: assay, morphoVariants: true}, {text: luciferase-assay, morphoVariants: true} | n/a | Linguamatics I2E |
| Experimental Validation | Reporter Assay | reporter-assay OR (reporter: {matchType: Substring} P/2 assay: {MatchType: Substring}) | {text: reporter-assay, morphoVariants: true} | n/a | Linguamatics I2E |
| Experimental Validation | ChIP | ChIP: {matchType: Substring} OR class: {snid: nlm.D047369, pt: Chromatin Immunoprecipitation} OR "Chromatin-Immunoprecipitation" | {text: ChIP, caseSensitive: true, matchType: Substring} | nlm->MESH 2021, nci-> NCI Derived 20.07d | Linguamatics I2E |
| Experimental Validation | CRISPR-Cas9 | CRISPR: {matchType: Substring} [optional: N/1 editing] OR Cas9: {matchType: Substring} OR CRISPR-Cas: {matchType: Substring} OR : {matchType: Substring} OR "clustered regularly interspaced short palindromic repeats" | default | n/a | Linguamatics I2E |
| Experimental Validation | Chromatin Looping Interaction | chromatin N/2 ((loop*: {matchType: Wildcard}) OR (interact*: {matchType: Wildcard})) OR chromatin-loop: {matchType: Substring} OR chromatin-interaction: {matchType: Substring} | default | n/a | Linguamatics I2E |
| Experimental Validation | TALEN | class: {snid: nlm.D000069896, pt: Transcription Activator-Like Effector Nucleases} | default | nlm->MESH 2021 | Linguamatics I2E |
| Experimental Validation | EMSA | class: {snid: nlm.D024202, pt: Electrophoretic Mobility Shift Assay} OR class: {snid: nci.C19362, pt: Electrophoretic Mobility Shift Assay} | default | nlm->MESH 2021, nci-> NCI Derived 20.07d | nlm->MESH 2021, nci-> NCI Derived 20.07d |
| Experimental Validation | MPRA | MPRA OR MPRAs OR "MPRA-": {matchType: Substring} | {text: MPRA, caseSensitive: true}, {text: MPRAs, caseSensitive: true}, {text: MPRA-, caseSensitive: true} | n/a | Linguamatics I2E |
| **Final Filter Strategy used:** | | filter: {Gene/Protein: any} AND filter: {Disease: any} AND filter: {**Publication Type**: “Research Article”} AND filter: { Non-coding Context: (enhancer OR insulator OR intergenic OR intragenic OR intron OR intronic OR miRNA OR non-coding OR promoter OR regulatory element OR silencer OR untranslated region)} AND filter: {Genetic Association: (Association OR Genetic Association Studies OR GWAS OR Locus)} AND filter: {Exp Validation: (Allele-specific OR ChIP OR Chromatin Looping Interaction OR CRISPR-Cas9 OR EMSA OR Luciferase OR Luciferase Assay OR MPRA OR Reporter Assay OR TALEN)} AND filter: {Functional Analysis: (Causal Variant OR Functional Variant OR Regulatory Variant OR rsID)} | | | |
